# Dysregulated Metabolism in People Living With HIV in the Modern ART‐Era: A Systematic Review of Targeted Metabolomics Studies

**DOI:** 10.1002/rmv.70179

**Published:** 2026-07-06

**Authors:** Levanco K. Asia, Du Toit Loots, Shayne Mason, Esme Jansen Van Vuren, Monray E. Williams

**Affiliations:** ^1^ Biomedical and Molecular Metabolism Research (BioMMet) North‐West University Potchefstroom South Africa; ^2^ Hypertension in Africa Research Team (HART) North‐West University Potchefstroom South Africa; ^3^ South African Medical Research Council Unit for Hypertension and Cardiovascular Disease North‐West University Potchefstroom South Africa

**Keywords:** antiretroviral therapy, HIV‐1, targeted metabolomics

## Abstract

HIV remains a significant public health issue, with 1.3 million new infections and 630,000 deaths annually, and 40.8 million people living with HIV (PLHIV) globally in 2024. Although antiretroviral therapy (ART) suppresses viral replication, it does not eradicate the virus, and metabolic dysregulation persists despite treatment. Metabolomics allows for a detailed investigation of these alterations; however, targeted metabolomics studies in ART‐treated PLHIV are limited, and there is no consensus on consistently dysregulated metabolites in PLHIV compared to healthy controls (HCs). This systematic review aimed to identify frequently investigated metabolites and key metabolic alterations in ART‐treated PLHIV. A search strategy was designed for this study. PubMed, Scopus, and Web of Science were searched to identify relevant articles. We collected all search results in a reference manager and assessed titles and abstracts, as well as the full‐text of the articles, for inclusion eligibility according to PRISMA guidelines. A total of 3217 studies were identified, of which 15 studies met the inclusion criteria. The review included 15 studies, comprising 886 PLHIV and 389 HCs. The most investigated metabolites were glutamine, tryptophan (Trp), kynurenine (Kyn), Kyn/Trp ratio, glycine, and ornithine. Consistent trends showed lower glutamine and glycine, and higher Kyn/Trp and ornithine, in PLHIV compared to HCs. Notably, metabolic dysregulation persisted in PLHIV despite viral suppression. Furthermore, studies conducted in the Global North more frequently reported metabolic dysregulation in PLHIV relative to HCs, compared with studies from the Global South, potentially reflecting regional variation or methodological differences. These findings offer insight into the targeted metabolic profiles of ART‐treated PLHIV using metabolomics and suggest that metabolites such as glutamine, glycine, Kyn/Trp ratio and ornithine may play important roles in understanding HIV‐1 pathogenesis in the modern ART‐era.

## 1 Introduction

Human immunodeficiency virus (HIV) affects approximately 40.8 million people globally [1, 2]. Eastern and Southern Africa is a highly endemic region for HIV, with approximately 20.5 million people living with HIV (PLHIV) located in these regions [1], accounting for more than half of the global HIV infection prevalence in this particular region. There is no cure for HIV at present, but there are effective management strategies such as antiretroviral therapy (ART). This has improved the quality of life of PLHIV to a point where the virus becomes undetectable [3]. However, only about 77% of PLHIV around the world are currently on ART [1] irrespective of the implementation of the diagnose‐and‐treat policies [4].

ART inhibits various steps in the HIV replication cycle, including: 1. Attachment, 2. Fusion, 3. Reverse transcription, 4. Integration, and 5. Protease‐mediated processing [5]. Hence, the use of ART stops the replication of HIV, but does not completely eradicate the virus, thereby making HIV infection a chronic disease. Additionally, the use of ART reduces HIV viral load to undetectable levels, making it untransmissible [5], allowing ART‐treated PLHIV to live relatively normal lives. However, a review by Capriotti [3] notes that ART use results in a variety of side effects, including metabolic complications [3]. These metabolic complications arise from dysregulated metabolism, defined as altered metabolite levels in PLHIV compared to healthy controls (HCs), caused by HIV infection or ART.

The metabolome is the complete set of small‐molecule metabolites within a biological sample [6]. The metabolome represents the closest downstream reflection of the genome and the functional phenotype of a cell or organism, and it can be altered by perturbations such as HIV infection and ART. The metabolome subsequently provides valuable insights into cell functionality and the consequential phenotype. These small metabolites in biological fluids can be quantified through a high‐throughput strategy known as metabolomics [6]. According to Collino, Martin [6], proton nuclear magnetic resonance (^1^H‐NMR) spectroscopy and mass spectrometry coupled to gas chromatography (GC‐MS) or liquid chromatography (LC‐MS), are the main analytical platforms used for metabolomics investigations [6]. Considering the diversity of metabolites and the various strengths and shortfalls of these analytical platforms, a combination of these tools can be useful in exploring the metabolome [7]. Additionally, metabolomics can employ both targeted and untargeted approaches. An untargeted approach provides broad coverage of metabolites, whereas a targeted approach enables accurate quantification of metabolites of interest. A targeted approach is therefore beneficial in diagnostics and/or for validating a potential biomarker measurement for disease.

The use of metabolomics in HIV research has allowed for the identification of those metabolites that are dysregulated during HIV infection [8]. A targeted metabolomics approach has been employed to accurately quantify specific metabolites or metabolite classes, enabling the assessment of their contributions to HIV pathogenesis and the underlying mechanisms of metabolic dysregulation [9]. Additionally, metabolomics provides insights into the role of ART, as its use results in a virally suppressed metabolic profile in PLHIV [8]. However, the metabolic side effects of ART have been shown to result in an increased risk for comorbidity in ART‐treated PLHIV [10]. Therefore, metabolic dysregulation may persist regardless of ART status. From the available evidence on this topic, there is no consensus as to which metabolites are commonly targeted in HIV‐1 studies, and further, no clear consensus as to which metabolites are persistently dysregulated in the modern ART‐era of HIV. To address this gap, we conducted a systematic review of the literature examining blood‐based (plasma/serum) metabolic profiles in ART‐treated PLHIV using targeted metabolomics. We aimed to identify commonly studied metabolites and to highlight potentially promising markers for future investigation.

## 2 Methods

### 2.1 Study Design

This is a narrative systematic review that summarises the existing literature on peripheral metabolic profiles in ART‐treated PLHIV using targeted metabolomics. This study has been carried out according to PRISMA guidelines [11] and the reporting of the study conforms to broad EQUATOR guidelines [12]. The study was registered on PROSPERO with the ID: CRD420250598485 (https://www.crd.york.ac.uk/PROSPERO/view/CRD420250598485).

### 2.2 Eligibility Criteria

Cross‐sectional and prospective studies were considered eligible if they included PLHIV receiving ART (no duration cut‐off) and included HCs as a comparator arm. Only studies reporting metabolite measurements from plasma or serum samples were included, as other biological matrices were outside the scope of this review. Plasma and serum were selected because they are minimally invasive and commonly used in metabolomics investigations. Studies were eligible if they used targeted metabolomics approaches to quantify metabolites using analytical platforms such as 1H‐NMR or MS coupled to LC, GC, HPLC. For the purposes of this review, targeted metabolomics was defined as the quantification of predefined metabolite targets using authentic reference standards and/or isotope‐labelled internal standards for metabolite identification and quantification. Both single‐metabolite assays and broader targeted metabolite panels were considered eligible, provided that metabolites were specifically targeted rather than profiled using untargeted discovery‐based approaches. There was no predefined minimum number of metabolites required for study inclusion. Consequently, studies investigating individual metabolites, amino acids, vitamins, micronutrients, or broader targeted metabolite panels were eligible if they fulfilled all other inclusion criteria.

Studies were excluded during title and abstract screening if they.

- were non‐primary research articles (including reviews, editorials, commentaries, hypotheses, book chapters, meta‐analyses, protocols, or retracted articles);
- did not investigate HIV‐1 infection in humans;
- focused on HIV‐2, simian immunodeficiency virus (SIV), feline immunodeficiency virus (FIV), plant metabolomics, or preclinical models (including cell culture, animal, in vivo, in vitro, or in silico studies);
- did not use targeted metabolomics approaches;
- investigated ART pharmacokinetics rather than host metabolism;
- involved pregnant participants.

Studies were excluded during full‐text screening if they.

- did not include an HIV‐negative HC comparator group;
- included PLHIV not receiving ART at the time of metabolomic assessment;
- did not use plasma or serum samples;
- used untargeted metabolomics or proteomics‐only approaches;
- involved participants with comorbidities or co‐infections that could substantially influence metabolic profiles, including but not limited to hepatitis B/C, tuberculosis, COVID‐19, cryptococcosis, cardiovascular disease (CVD), diabetes, hypertension, liver cirrhosis, malignancies, obstructive lung disease, nephrotoxicity, or mental health disorders;
- involved > 25% of participants who were active smokers, alcohol users, recreational drug users as well as studies that are ambiguous and do not report on the percentage or number of smokers present in the cohort;
- involved vaccinated participants, pregnant participants, or individuals younger than 18 years of age; or
- did not clearly report HIV status or participant group allocation.

Where broader studies contained subgroup comparisons meeting all predefined eligibility criteria, only data from the eligible subgroup were extracted and included in the synthesis. For example, where studies included participants with substance use, supplementation interventions, or comorbidities, only the eligible subgroup comparisons without these confounding factors were considered for extraction and analysis.

Non‐English publications, conference abstracts, and grey literature were excluded, as previous evidence suggests that exclusion of such studies is unlikely to substantially influence findings in health‐related systematic reviews [13, 14]. Therefore, only indexed peer‐reviewed studies were included. A detailed breakdown of study exclusions is presented in Section 3.1.

### 2.3 Data Sources

**FIGURE 1:**
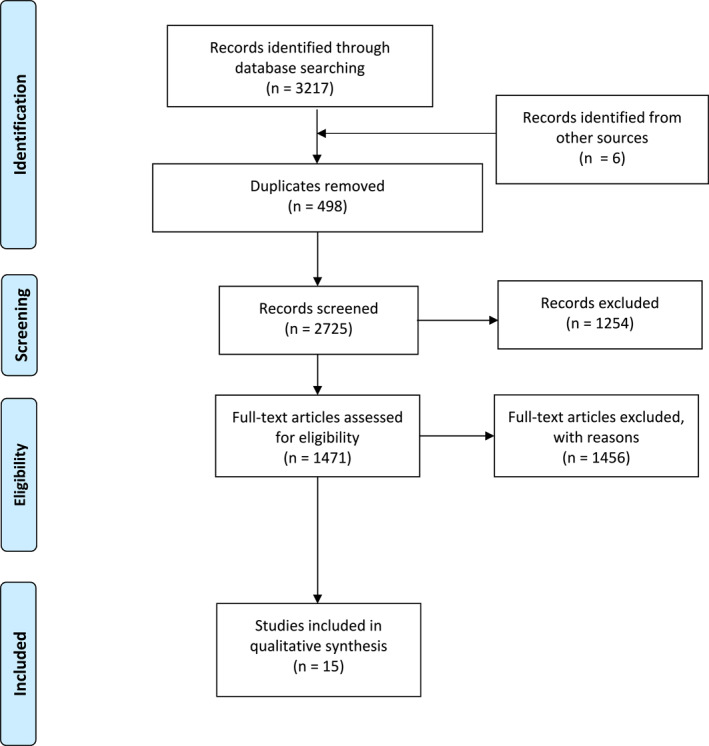
PRISMA flow diagram.

We searched a variety of databases (PubMed, Scopus, and Web of Science) for eligible articles on the 27th of August 2025. The searches were done without any publication date limits. PubMed's search terms included (Human immunodeficiency virus [tw] OR Human immunodeficiency virus [mh]) AND (liquid chromatography [tw] OR chromatography, liquid [mh] OR Gas chromatography [tw] OR Chromatography, Gas [mh] OR nuclear magnetic resonance [tw] OR magnetic resonance spectroscopy [tw] OR Magnetic Resonance Spectroscopy [mh] OR mass spectrometry [mh] OR tandem mass spectrometry [mh] OR targeted metabolomics [tw] OR metabolomics [mh]) AND (blood [tw] OR blood [mh] OR serum [tw] OR plasma [tw]). The full search terms applied to each database are provided in (Supporting Information S1). The search terms were focused on HIV, metabolomics, ART, and blood (plasma and/or serum) samples. Additionally, the search strategy and retrieved articles are illustrated in Figure 1.

### 2.4 Data Selection

All articles were retrieved and loaded onto a single database using a reference manager (EndNote X9, Clarivate, PA, USA). Two authors (LA and MW) independently identified studies meeting the inclusion criteria. If there were any discrepancies in article inclusion/exclusion, these were discussed among all authors (LA, MW, EJvV, SM, and DL), and a decision was reached regarding the suitability of the article for inclusion or exclusion based on the predefined criteria (Section 2.2). Deduplication was conducted using a two‐step approach. First, records from PubMed, Scopus, and Web of Science were initially collated in Microsoft Excel, where duplicate titles were identified through sorting and conditional formatting and subsequently manually verified. The dataset was then imported into EndNote (version 21), and the ‘Find Duplicates’ function was applied to identify any remaining duplicates based on author, year, and title fields. All identified duplicates were manually reviewed prior to removal.

Studies for which full texts were not immediately accessible were initially retained during screening to avoid premature exclusion of potentially relevant articles. Following title and abstract screening, studies meeting exclusion criteria at the preliminary stage were removed prior to full‐text retrieval attempts. For studies requiring full‐text assessment but unavailable through institutional access, additional retrieval efforts were undertaken through institutional library services, interlibrary loan requests, and direct author contact. Corresponding authors were contacted first, and where corresponding author information was unavailable, publicly listed co‐authors were contacted. Authors were given a 2‐week period to respond before studies were classified as inaccessible and excluded from the review. A detailed list of the criteria is described in Section 3.1.

### 2.5 Quality Assessment of Included Studies

We used kappa statistics and the Joanna Briggs Institute (JBI) critical appraisal tools to assess the quality of the studies included in this review. We adopted the JBI tools by incorporating a Likert scale [15] to provide a quantitative measure of study quality, as done previously [16, 17]. For the evaluation, we focused on the JBI quality questions from the “Checklist for Analytical Cross‐Sectional Studies” that could influence the findings. These questions addressed (1) the definition and description of cohort information, (2) the validity and conditions of measurement (e.g., viral load and CD4 count), and (3) consideration of confounding factors and statistical analysis.

Specifically, we assessed all studies according to the following questions (1) Was the cohort information (i.e., setting, sample type, participants, ART use and regimen, etc.) clearly described? (2) Was the method (targeted, the use of internal standards, solvents, columns, mobile phases, protein precipitation, etc.) for quantifying the metabolic profile's objective, valid, reliable, and properly described? and (3) Did the study consider potential confounding factors for example, lifestyle factors, and employ appropriate statistical analysis to account for the identified potential confounders?

Each criterion was rated as 0 = no, 1 = partly, or 2 = yes. Those studies that complied with all the aforementioned standards and had a total rating of ≥ 5 were classified as high quality; those with a rating between 3 and 4 were considered intermediate quality; and those with a rating of ≤ 2 were classified as low quality (see Table S1).

### 2.6 Potential Confounders: Sex, Age, CD4 Count, Viral Load, ART, Geographic Location, BMI and Diet/Fasting State

Several factors may potentially influence the metabolic profiles in the studies and thus the interpretation of the findings and results of this review. These factors include sex [18], age [19], CD4 count [20], viral load [21], ART [8], geographic location, BMI, and fasting state. To account for these potential confounders in the interpretation of this review, we stratified studies by “high” and “low” for each variable and assessed whether a trend in metabolic profiles was present in each study. For this sub‐analysis, we considered metabolites investigated in two or more independent studies.

We examined the sex distribution of participants in the included studies. If more than 70% of participants in a study were of one sex, the study was categorised as predominantly representing that sex (e.g., if over 70% of participants were male, the study was classified as predominantly male for stratification purposes). Studies in which neither sex exceeded 70% representation were categorised as mixed‐sex cohorts.

Further, we stratified studies by PLHIV age to determine whether age influences dysregulated metabolism in PLHIV. Here, we stratified PLHIV by age group (years): 30–39, 40–49, and 50–59. Notably, no studies reported participant age groups younger than 30 years or older than 60 years, limiting additional age‐based stratification for these groups.

We investigated CD4 counts and stratified participants into low (< 200 cells/mm^3^) and high (> 200 cells/mm^3^) groups. Next, we investigated viral load and stratified the study population according to viral load: undetectable/viral suppression (< 50 copies/mL) and detectable (> 50 copies/mL) [22].

Next, regional variations in HIV can influence inflammation in PLHIV, and given the immune–metabolic interactions, it could also affect metabolite levels. Thus, using the reported geographical locations of the participants, we stratified studies into the Global North and the Global South to determine whether this influences metabolic profiles. Although stratification by HIV subtype was initially considered, this information was not reported in the included studies. Alternative epidemiological stratifications, including geographical region and ethnicity/race, were therefore explored. However, ethnicity was inconsistently reported across studies, and studies that did report ethnicity often included heterogeneous participant populations, limiting meaningful subgroup comparisons. Consequently, only geographical region (Global North vs. Global South) was investigated.

Non‐viral factors, including BMI and fasting state, were inconsistently reported across studies and therefore could not be formally stratified. Where available, these variables were extracted and summarised descriptively. Where stratification was not feasible because of limited reporting or restricted variability across studies, this was stated in the Results section.

## 3 Results

### 3.1 Study Characteristics

A total of 3217 records were collated from database searches in a reference manager and assessed for eligibility (Section 2.2). An additional six studies were identified from other sources. After removing 498 duplicates, 2725 titles and abstracts were screened, as shown in Figure 1, leaving 1471 articles for full‐text assessment. Following full‐text screening, 1456 articles were excluded for the reasons detailed below, resulting in 15 studies included in the final review.

Exclusions included reviews, book chapters, editorials, hypotheses, commentaries, meta‐analyses, retracted articles, protocols, safety procedures, and study/cohort designs (*n* = 29); non‐English language (*n* = 1); studies not focused on HIV (*n* = 31); no HIV‐positive participants (*n* = 60); use of routine laboratory techniques rather than metabolomics (*n* = 49); ART pharmacokinetics/pharmacology studies (*n* = 548); untargeted metabolomics (*n* = 19); sample types other than plasma and/or serum (*n* = 304); absence of HIV‐negative comparison groups (*n* = 58); unreported, negative, or unclear ART status among PLHIV (*n* = 42); participant (> 25%) drug use, smoking, or alcohol use (*n* = 14); comorbidities (*n* = 85); pregnancy and/or age under 18 years (*n* = 36); preclinical studies (*n* = 52); proteomics or lipidomics analyses (*n* = 36); unreported HIV status of sample types (*n* = 12); and use of other medications or vaccinations (*n* = 10). Finally, 54 studies were initially categorised as unavailable for full‐text assessment. After refined title and abstract re‐screening, 41 were excluded because they met preliminary exclusion criteria and did not require full‐text retrieval. The remaining 13 studies underwent additional retrieval efforts, including institutional library searches, interlibrary loan requests, and direct author contact. Corresponding authors were contacted first; where this information was unavailable, other publicly listed co‐authors were contacted. Authors were given 2 weeks to respond. One author provided the requested full text for eligibility assessment. Despite these efforts, the remaining studies could not be accessed and were excluded from the final review.

After excluding articles based on the eligibility criteria (Section 2.2), a total of *n* = 15 were included for data extraction. Cohort information was extracted from all included studies (Table 1). In total, there were *n* = 886 PLHIV and *n* = 389 HCs across the studies, although two studies did not report the number of HCs [28, 80]. Age was reported for both PLHIV and HCs in all studies except three, which did not provide age data for HCs [25, 28, 30]. The mean age was 41.15 years for PLHIV and 34.6 years for HCs. All studies reported sex, while only 10 provided geographic location: Global North (*n* = 5) and Global South (*n* = 5).

**TABLE 1:** Cohort information of PLHIV and HCs in the included studies.

Reference	Geographical location	ART status reported	PLHIV and HC groups	Number of participants (n)	CD4+ count (cells/mm^3^)	Viral load (copies/mL)	Age (years)	Sex, male/female (%)
[23]	Brazil	Yes	PLHIV	71	207 (116–454) 49% of participants had CD4 counts less than 200	N/D	30 ± 7.2	35%/65%
			HCs	25	805 (657–870) 4% of participants had CD4 counts less than 200		23 ± 6.6	36%/64%
[24]	USA	Yes	PLHIV	30	256 (114–395)	36,491 (6795–124 622)	52 (43–59)	76.6%/23.4%
			HCs	28	N/D		40 (33–48)	N/D/N/D
[25]	South Africa	Yes	PLHIV	12 on ART	413.33	N/D	N/D	N/D/N/D
			HCs	11	N/D		N/D	N/D/N/D
[26]	Sweden	Yes	PLHIV	64 (PLHIV ART group) 55 (For plasma targeted metabolomics	605 (490–777)	N/D	50 (9.3)	68.8%/31.2%
			HCs	37 37 (For plasma targeted metabolomics)	N/D		49.4 (9.6)	37.8%/62.2%
[27]	N/D	Yes	PLHIV	35	232 ± 224 [20–1000] (All PLHIV) 83 ± 55 [20–200] (PLHIV < 200 cells/mm^3^ group)	N/D	39 ± 7 [24–54]	86%/14%
			HCs	35	N/D		33 ± 7 [22–53]	66%/34%
[21]	USA	Yes	PLHIV	11	195 (128)	145,000 (214,000)	44 [26–64]	82%/18%
			HCs	N/D	N/D		N/D	N/D/N/D
[28]	Brazil	Yes	PLHIV	21	421.3 (170.6)	50% had < 80	38 [29–59]	N/D/N/D
			HCs	N/D	N/D		N/D	N/D/N/D
[29]	USA	Yes	PLHIV	205	512 (30, 2135) *Nadir CD4 count was 143 (0, 668)*	< 50	52 (35, 87)	81%/19%
			HCs	99	859 (360, 1717) *Nadir CD4 count was* 581 (242, 1198)		52 (35, 83)	81%/19%
[29]	China	Yes	PLHIV	76	248 (121–297) at ART initiation/baseline	After 1 year on HAART: < 40 in 94.7% participants	32 (28–39.5)	76.3%/23.7%
			HCs	16	N/D		32 (27–47)	62.5%/37.5%
[30]	China	Yes	PLHIV	127	403 (332–560) on ART	< 20	32 (27–44) at baseline/ART initiation	92.1%/7.9%
			HCs	25	N/D		N/D	N/D/N/D
[31]	Canada	Yes	PLHIV	88 of the successfully treated (ST)	531.3 ± 267.4 [37–1282]	< 39.81	48.4 ± 9.4	83%/17%
			HCs	50	812.6 ± 273.0 [281–1559]		45.0 ± 9.9	70%/30%
[33]	USA	Yes	PLHIV	24 in the INR group 15 in the IR group	INR: 229 (155–279) IR: 628 (532–1011) *Nadir CD4 count were* *21 (7‐56) for the INR group and 105 (49‐311) for the IR group*	Undetectable	INR: 47 (43–52) IR: 49 (47–55)	INR: 100%/0% IR: 93%/7%
			HCs	19	N/D		47 (37–55)	100%/0%
[34]	China	Yes	PLHIV	38	357 (264–527)	Undetectable	32 (26–50)	89.47%/10.53%
			HCs	18	N/D		31 (26–41)	66.67%/33.33%
[35]	N/D	Yes	PLHIV	35 in the INR group 33 in the IR group	INR: 195 (99–318) IR: 656 (417–1257)	Undetectable	INR: 44 (31–77) IR: 31 (25–49)	INR: 91%/9% IR: 100%/0%
			HCs	16	682 (427–1031)		30 (23–36)	94%/6%
[36]	USA	Yes	PLHIV	10 with no stimulant use	N/D	Undetectable	38 (± 12)	100%/0%
			HCs	20 with no stimulant use	N/D		36 (± 15)	100%/0%

*Note:* Median (IQR); median ± SD; median (min, max); mean (SD); mean [range]; mean ± SD [range]. *Disclaimers*: A subset of the cohort was used for metabolomics work but the data for this subset is not available regarding ages, sex of the subset cohort (Sitole et al., 2019); Controls were from two normal controls from their lab and from two previously cited studies – One of the studies report that the participants were on lipid lowering agents whereas the other study participants were not on any lipid lowering agents (McRae et al., 2010) [21]; the sex of the participants were not well described (Neves et al., 2006) [28]. Effect sizes were reported without accompanying *p*‐values (Gebremicael et al., 2019; McRae et al., 2010; Sitole et al., 2019). These findings should be interpreted with caution.

Abbreviations: HCs: healthy controls; INR: immunological non‐responders; IR: immunological responders; N/D: not described; PLHIV: people living with HIV; ST: successfully treated; USA: United States of America.

### 3.2 Quality Assessment of the Included Studies

Two authors, LA and MW, assessed and rated the quality of the included studies. The Kappa statistic for interrater agreement and reliability was 0.59, indicating moderate‐to‐substantial agreement [81]. Most studies were rated as intermediate to high quality. Depending on the reviewer, high‐quality accounted for 40%–47% of assessments, while intermediate‐quality accounted for 53%–60%. None of the selected studies was rated as low quality by either reviewer. Therefore, based on the quality rating of the included studies, recommendations were made in the latter part of this review.

### 3.3 Sample Matrix

This systematic review specifically included articles that utilised targeted metabolomics on serum and plasma of ART‐treated PLHIV. Thirteen studies investigated plasma metabolite profiles [23, 24, 26, 27, 29, 30, 31, 32, 33, 34, 35, 36, 80], whereas one study investigated serum metabolite profiles [25]. Additionally, one study used the terms ‘plasma’ and ‘serum’ interchangeably, and thus, it was not clear which sample type was used and was kept since the metabolome extracted from either will be inherently similar and is both representative of blood [28] (Table 2).

**TABLE 2:** The metabolic profiles of PLHIV and HCs in the included studies.

Reference	Sample type	Metabolic profile/s investigated	Metabolomics analytical platform	Quantification of metabolic profiles	Other key findings
[23]	Plasma/serum	Vitamin A	High performance liquid chromatography attached to ultraviolet detector (HPLC‐UV)	Vitamin A was higher in PLHIV (1.78 (1.15–1.99) μmol/L) compared to HCs (1.5 (1.1–1.6) μmol/L), however, no statistical analysis was reported to assess whether this difference was significant.	**1. Vitamin A levels at baseline:** **PLHIV** versus **HCs** 1.4 (1.1–1.7) versus 1.5 (1.1–1.6), respectively (*p =* 0.8588). **2. Vitamin A levels for PLHIV eligible** versus **ineligible for initiating HAART Baseline:** 1.55 (1.40–1.95) versus 1.33(1.08–1.66), respectively (*p =* 0.0715). **At 6‐month follow‐up:** 1.78(1.15–1.99) versus 1.78(1.64–1.85), respectively (*p* = 0.7655). 66.2% of PLHIV were classified as having normal nutrition compared to 88% of HCs. The remaining participants either had severe, moderate, mild nutrition or were considered overweight.
[24]	Plasma	Lysophosphatidic acid (LPA), LPA 18:2	LC‐MS included a Agilent 1200‐HPLC system AND Selected reaction monitoring (SRM) mass spectrometry	LPA subtype, LPA 18:2, was elevated in PLHIV (a subgroup of *n* = 5) at the start of ART compared to HCs (*p* < 0.05). This persisted at 24 weeks of ART but was not significantly higher compared to the HCs (*p* > 0.05).	Autotaxin and immune markers did not correlate. sCD14 levels correlated with Mac2 Binding protein (Mac2BP) (*p* = 0.039). IL‐6 and sCD163 levels decreased to near normal (comparable to the HCs) after initiating ART (*p =* 0.05 and *p =* 0.009, respectively).
[25]	Serum	Selection criteria for analysis: 1. Metabolites must be within the limits of detection 2. Metabolites must be associated with HIV/cART‐induced oxidative stress Therefore, the metabolites investigated were: Alanine Aspartic acid Glutamic acid Glutamine Phenylalanine Proline Tryptophan Tyrosine Cystine	GC‐MS	**Higher levels of metabolic profiles in PLHIV compared to HCs (fold changes):** Alanine (2.64) Aspartic acid (2.4) Glutamic acid (2.2) Phenylalanine (2.65) Tyrosine (1.21) **Lower levels of metabolic profiles in PLHIV compared to HCs (fold changes):** Glutamine (0.51) Proline (0.73) Tryptophan (0.49) Cystine (0.05)	**1. OPLS‐DA model:** Perfect classifier and statistically reliable, very good predictive capability as there were no signs of any overfitting (indicated by cross validation). **2. OPLS‐DA loading plot:** Identified potential discriminant features such as variables that combined high covariation and correlation. **3. VIP:** VIP scores were further used as a validation checkpoint. Elevated glutamic acid and cystine is speculated to contribute to oxidative stress and/or onset of HIV‐dementia.
[26]	Plasma	Central carbon metabolism (CCM) Amino acids	CCM: GC‐MS Amino acids: LC‐MS/MS	**PLHIV on ART** versus **HCs (adjusted *p* *<* 0.05):**Increases in amino acids: Ornithine Glutamate Decrease in Amino acids: Methionine Lysine Tryptophan Glutamine Glycine Threonine Arginine Kynurenine Increases in the CCM and sugar metabolites: Lactate Pyruvate Fumarate *β*‐Glucose Oxoglutarate Decreases in the CCM and sugar metabolites: Maltose Citrate Glycerate Glucose	**1. UMAP analysis:** Alterations in CCM and sugar metabolites are associated with the duration of ART treatment.
[27]	Plasma	Total glutathione Glutathione (non‐protein‐bound) Total thiols Thiobarbituric acid‐reactive substances (TBARS)	HPLC HPLC Fluorescence method	**Total glutathione:** Lower in PLHIV compared to HCs (95% CI, 0.362–1.690; *p* = 0.003). Significantly lower in PLHIV CD4 < 200 group compared to the HCs (95% CI, 0.676–2.313; *p* = 0.001). **Non‐protein‐bound glutathione:** No difference in PLHIV and HCs **TBARS:** No difference in PLHIV and HCs **No difference in PLHIV and HCs (Total thiols):** Cysteine Cysteinylglycine Glutamylcysteine Homocysteine	No additional relevant findings.
[21]	Plasma	Bile acids: Cholic acid (CA) Chenodeoxycholic acid (CDCA) Deoxycholic acid (DCA) Lithocholic acid (LCA) Ursodeoxycholic acid (UDCA) Taurocholic acid (TC)	LC‐MS/MS	Mean LCA were higher in PLHIV compared to HCs, 15.8–36.3 ng/mL compared to 1.1–7.5 ng/mL), respectively. Mean TC were higher in PLHIV compared to HCs, 105.2–429.7 ng/mL compared to 0–128.5 ng/mL, respectively. CA, CDCA, UDCA, and DCA were not different between PLHIV and HCs. Bile acid concentrations were similar with both the dual and triple protease inhibitor therapy for all patients. No statistical analysis was used to compare significant differences between groups.	**1. Mean intrapatient variability (PK2‐PK3) in AUC:** CA = 26.9% CDCA = 34.3% DCA = 31.8% LCA = 50.3% UDCA = 42.6% TC = 44.3% **2. Interpatient variability ranges in PK1‐PK3 for PLHIV:** CA = 53–84% CDCA = 49–92% DCA = 79–94% LCA = 108–157% UDCA = 48–60% TC = 109–215% CA, DCA, UDCA interpatient variability was lower in PLHIV compared to HCs which had CA of 103%–115%, DCA of 120%–125%, UDCA of 79%. CDCA and TC interpatient variability was similar in PLHIV compared to the HCs with CDCA of 62%–84% and TC of 110%. LCA interpatient variability was higher in PLHIV compared to HCs, 108%–157% compared to 50%. **3. Relative bile contribution:** **PLHIV:** CDCA = 9% DCA = 34% UDCA = 17% CA = 5% TC = 28% LCA = 6% **HCs:** CDCA = 37% DCA = 18% UDCA = 7% CA = 22% TC = 3% LCA = 4% **Previously cited HCs:** CDCA = 31–50% DCA = 20–37% UDCA = 18% CA = 13–17% TC = 11% LCA = 2%
[28]	Plasma/serum	Retinol	HPLC with multiwavelength detection	PLHIV with no vitamin replacement had a significant decrease in serum retinol levels from 1.77 µmol/L to 1.55 μmol/L, *p* = 0.017, however, no statistical analysis was used to compare significant differences between groups.	No other findings on participants that were not on vitamin supplementation.
[29]	Plasma	Kynurenine/Tryptophan (Kyn/Trp) ratio	HPLC (detection method not reported)	Kyn/Trp was higher in PLHIV (*p* < 0.0001).	Age was associated with increased Kyn/Trp (*p* < 0.0001). CD4 count and CD4 nadir count did not have any relationship with the Kyn/Trp ratio. LPS increased when Kyn/Trp ratio decreased but had no relationship with HCs (*p* = 0.0071). Kyn/Trp activity and neopterin were positively associated in PLHIV and HCs.
[29]	Plasma	Trp Kyn Kyn/Trp ratio	HPLC with wavelength ultraviolet detection	Trp and Kyn in the PLHIV were comparable to that of the HCs (not significant). Kyn/Trp ratio in PLHIV became near normal compared to HCs (*p* < 0.05).	**1. After 1 year on HAART:** CD4 counts did not correlate with Trp, Kyn, and Kyn/Trp. Plasma sCD14 did not correlate with Kyn/Trp in both the PLHIV (*p* = 0.277) and HCs (*p* = 0.368).
[30]	Plasma	Kyn/Trp	Ultra‐performance LC‐MS	Kyn/Trp decreased significantly in PLHIV but was still higher compared to HCs (*p* < 0.05).	**1. On ART HIV DNA is significantly associated with the following:** Inverse prediction with pre‐ART CD4 T‐cell count (*r* = −0.27, *p* = 0.003). Inverse prediction with pre‐ART CD4/CD8) (*r* = −0.41, *p* < 0.0001). Positive prediction of pre‐ART CD8 T‐cell (*r* = 0.22, *p* = 0.013). Inversely correlated with ART duration (*r* = −0.38, *p* < 0.0001). Correlated with on‐ART CD8 T‐cell counts (*r* = 0.32, *p* = 0.0003). Correlated with on‐ART CD4/CD8 ratio (*r* = −0.41, *p* < 0.0001). **2. Multivariate binary logistic regression model:** Low pre‐Art CD4/CD8 and high on‐ART Kyn/Trp were independently associated with high on‐ART HIV DNA (higher than median) (*p* = 0.003 and *p* < 0.0001, respectively).
[31]	Plasma	Trp Kyn 3‐Hydroxykynurenine (3‐OH‐Kyn)	Automated online solid‐phase extraction‐liquid chromatographic‐tandem mass spectrometric method (XLC‐MS/MS)	Kyn levels were significantly higher in PLHIV compared to HCs (*p =* 0.0079). Kyn/Trp levels were significantly higher in PLHIV compared to HCs (*p* = 0.0020). No difference between PLHIV and HCs for Trp and 3‐OH‐Kyn.	In the entire cohort, Kyn/Trp ratio associated with Th17/Treg ratio (*p* = 0.0435) and Treg frequency (*p* = 0.0034).
[33]	Plasma	Kyn/Trp	LC‐MS/MS	Kyn/Trp was higher in PLHIV compared to HCs (*p* ≤ 0.04).	Higher degree of mucosal apoptosis was positively associated with Kyn/Trp (*p* = 0.08).
[34]	Plasma	Trp Kyn Kyn/Trp	HPLC	Trp was significant lower in PLHIV than in HCs (*p* < 0.05). Kyn/Trp and Kyn in PLHIV were nearly similar to HCs.	**1. After** **ART** **use:** CD4 cell count did not differ significantly (*p* = 0.117). Changes in Kyn/Trp and Kyn were positively correlated with concentrations of sVCAM‐1, sICAM‐1, and Ang‐II.
[35]	Plasma	Amino acids Amino acid derivatives Peptides/small peptides Bile acids Porphyrin/heme metabolites Organic acids & sugar acids Phenolic compounds/polyphenols Fatty acids & fatty acid derivatives TCA cycle intermediates/energy metabolites Nucleotides & nucleosides Sulphated/phosphorylated metabolites Steroids & vitamin derivatives Carnitines/acylcarnitines Maillard reaction products (glycated amino acids/AGEs) Creatine/renal markers Amino sugars Neurotransmitters/signalling molecules Fatty acid amides (endocannabinoid‐like) Phospholipids/glycerophospholipids Microbiome‐derived metabolites Sphingolipids Alkaloids & heterocycles Plant‐derived metabolites	Agilent 1290 II UPLC coupled to Sciex 5600+ quadrupole‐TOF MS	**Metabolites were different between PLHIV INRs and HCs (p < 0.05) in the following classes:** Amino acids Amino acid derivatives Peptides/small peptides Bile acids Porphyrin/heme metabolites Organic acids & sugar acids Phenolic compounds/polyphenols Fatty acids & fatty acid derivatives TCA cycle intermediates/energy metabolites Nucleotides & nucleosides Sulphated & phosphorylated metabolites Steroids & vitamin derivatives Plant/xenobiotic metabolites Carnitines (acylcarnitines) Maillard reaction products (AGEs, glycated amino acids) Creatine/renal markers Amino sugars Neurotransmitters Fatty acid amides (endocannabinoid‐like) Phospholipids/glycerophospholipids Microbiome‐derived metabolites Sphingolipids Alkaloids & heterocycles **Metabolites were different between the PLHIV IRs and HCs for the following classes (*p* < 0.05):** Amino acids Amino acid derivatives Peptides/small peptides Bile acids Porphyrin/heme metabolites Organic acids & sugar acids Phenolic compounds/polyphenols Fatty acids & fatty acid derivatives Nucleotides & nucleosides Sulphated/phosphorylated metabolites Steroids & vitamin derivatives Carnitines/acylcarnitines Neurotransmitters/signalling molecules Phospholipids/glycerophospholipids Xenobiotics/plant‐derived metabolites Sphingolipids Alkaloids & heterocycles *Disclaimer: An extensive list of all the* specific *metabolites are listed in supplementary file 2.*	**CD4/CD8 ratio, a biomarker for immune recovery and non‐AIDS related events, positively correlated** **with the following metabolites:** 16 alpha‐hydroxy DHEA 3‐sulphate Cysteineglutathione disulfide O‐phosphoethanolamine Phosphorylcholine Thymine **CD4/CD8 ratio negatively correlated with:** 3‐Hydroxyoctanoic acid **The following metabolites were positively correlated with inflammation markers:** 16 alphahydroxy DHEA 3‐sulphate 3‐Hydroxyoctanoic acid O‐phosphoethanolamine **The following metabolites negatively correlated with inflammation markers:** Phosphorylcholine Thymine
[36]	Plasma	Biocrates MxP Quant 500 Kit using stable isotope labelled internal standards. *Disclaimer: The specific metabolites investigated are not well described.*	LC‐MS/MS	**There were not statistical differences between PLHIV with no stimulant use and HCs with no stimulant use (*p* > 0.05):** Choline Tryptophan	There are no other findings between the PLHIV with no stimulant use and the HCs with no stimulant use.

*Note:* Controls were from two normal controls from their lab and from two previously cited studies – One of the studies report that the participants were on lipid lowering agents whereas the other study participants were not on any lipid lowering agents [21]. Plasma and serum were used interchangeably [23, 28].

### 3.4 Metabolomics Platform and Detection

From all studies, six used HPLC but were coupled to different detectors, such as UV [23], fluorescence [27], multiwavelength detection [28], wavelength UV detection [32], and MS [34]. For one study, the detection method was not reported [29]. Next, three studies used LC‐MS/MS [33, 36, 80]. Also, only one study used GC‐MS [25], UPLC [30], XLC‐MS/MS [31], and a UPLC coupled to a TOF/MS [35]. Additionally, one article used both LC‐MS and SRM‐MS [24]. Lastly, one study used both GC‐MS and LC‐MS/MS [26]. None (0%) of the included studies utilised ^1^H‐NMR to investigate targeted metabolomics profiles for PLHIV (Table 2). There is heterogeneity with the reporting of analytical instrumentation, reference standards, internal standards, protein removal techniques, and extraction methods (Table S2). Additionally, not all studies reported on the limit of detection, limit of quantification, and the presence of batch effects or if batch corrections were done (Table S2).

### 3.5 Metabolic Profiles Investigated

A wide range of metabolites was investigated across the included studies (supplementary file 2), with several metabolites analysed in more than one study. The Kyn/Trp ratio was investigated in six studies [29, 30, 31, 32, 33, 34] (Table 2); kynurenine (Kyn) in four studies [26, 31, 32, 34]; tryptophan (Trp) in six studies [25, 26, 31, 32, 34, 36]; and glutamine [25, 26], glycine [26, 35], and ornithine [26, 35] in at least two independent studies. The remaining metabolites were only investigated in a single study (Table 2).

**FIGURE 2:**
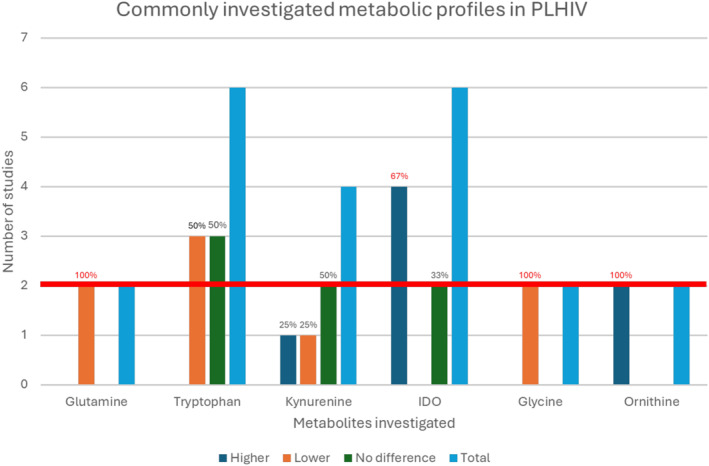
The frequency of metabolic profiles investigated. The metabolites that were commonly investigated are shown on the *x*‐axis. The light blue indicates the total number of studies investigating the metabolic profile. The dark blue, orange, and green bars represent studies reporting high, low, and no difference in metabolite levels in ART‐treated PLHIV compared to HCs, respectively. A metabolic profile was considered noteworthy if 2 or more independent studies investigated it and is represented by the red cut‐off line – criterion 1. The percentage above a bar shows that a metabolic profile was consistently reported as high, low, or no difference in more than 60% of the studies indicated in red text – criterion 2. Effect sizes were reported without accompanying *p*‐values in some studies [23, 25, 80].

Certain metabolites were investigated more frequently than others. Therefore, when interpreting the findings, we considered the frequency with which metabolites were investigated. Thus, we adapted and applied previously established criteria to identify “noteworthy” markers/findings [16, 17, 37]. Subsequently, we considered a metabolite to be “noteworthy” if (1) it was investigated in at least two independent studies (≥ 2) (red cut‐off line; Figure 2), and (2) more than 60% of the studies reported a consistent trend in its metabolite levels in PLHIV compared to HCs. In other words, if over 60% of studies investigating a particular metabolite found consistent directional changes (lower, higher, or no difference in PLHIV compared to HCs) in its levels associated with HIV‐1 infection in the presence of ART, it was identified as a noteworthy candidate for future research.

**FIGURE 3:**
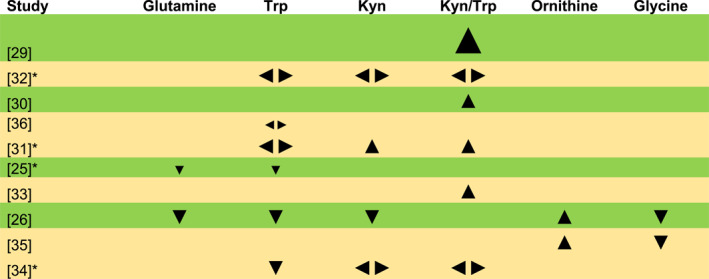
Effect Plot of most investigated metabolites. The effect plot illustrates the direction of dysregulation for metabolites investigated in two or more independent studies, including glutamine, tryptophan (Trp), kynurenine (Kyn), Kyn/Trp, ornithine, and glycine. Effect direction: ▴ indicates upregulation; ▾ indicated down regulation; ◄► indicates no difference. Sample size: The total sample size of participants that targeted metabolomics was done on are indicated by the size of the arrows with a large arrow ▴> 300 participants; a medium arrow ▴ 50‐300 participants; a small arrow ▴< 15 participants. *Indicates studies that did not report *p*‐values but provided alternative statistical evidence supporting the reported directional findings.

From the included studies, glutamine, Trp, Kyn, Kyn/Trp, glycine, and ornithine (Table 2) were investigated by at least 2 independent studies (indicated by a red cut‐off line; Figure 2), thus meeting criterion 1. Subsequently, we assessed where studies (> 60%) reported a consistent trend in their levels in PLHIV (criteria 2, Figure 2). More than 60% of studies investigating glutamine, Kyn/Trp, glycine, and ornithine reported consistency in their levels, which included lower glutamine, higher Kyn/Trp, lower glycine, and higher ornithine in PLHIV compared to HCs, thus meeting criterion 2 (Figure 2). The metabolic profile of Trp and Kyn had mixed findings (Figure 2). Furthermore, it should be noted that three studies did not report on *p*‐values when comparing metabolic profile levels between PLHIV and HCs [23, 25, 80]. These studies were still included because they compared metabolic levels in PLHIV and HCs, but were limited by the statistical analyses. Therefore, these three studies should be interpreted with caution. In addition, we included an effect plot to visually summarise the direction of dysregulation of metabolites investigated in two or more independent studies while also accounting for study sample size. Trp and Kyn demonstrated mixed findings across studies. In contrast, glutamine and glycine were consistently reported to be lower in PLHIV compared with HCs in 100% of studies investigating these metabolites. Similarly, Kyn/Trp and ornithine were consistently reported to be higher in PLHIV in 67% and 100% of studies, respectively. A sample‐size weighting analysis was additionally conducted to contextualise the relative contribution of individual studies to the compiled findings (Figure 3). These findings should therefore be interpreted with caution, as the relative weight of evidence differs across metabolites and studies.

### 3.6 Potential Confounders: Sex, Age, CD4 Count, Viral Load, ART, Geographic Location, BMI and Diet/Fasting State

**FIGURE 4:**
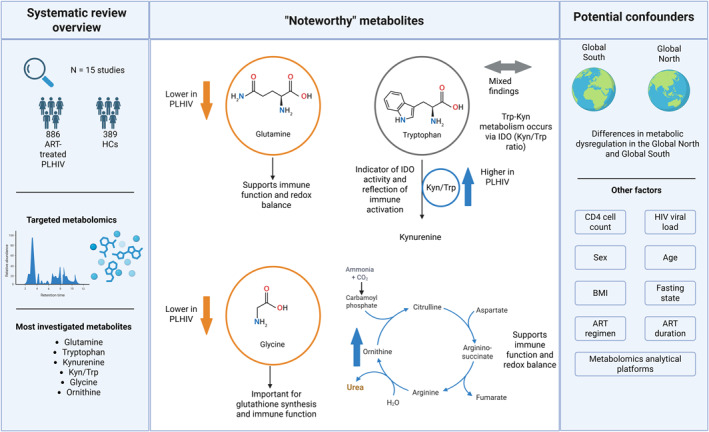
Graphical summary of key findings. The 15 studies (886 ART‐treated PLHIV; 389 HCs) shows a consistent metabolic pattern in PLHIV: lower (indicated by orange arrows) glutamine and glycine, and higher (indicated by blue arrows) Kyn/Trp ratio and ornithine. These changes indicate altered amino acid metabolism, increased Trp catabolism, and urea cycle dysregulation. Notably, these alterations persist despite ART, high CD4 counts, and undetectable viral loads. Metabolic dysregulation and differences are shown in the Global North and Global South, potentially reflecting HIV subtype variation and/or non‐viral factors such as BMI, fasting state, and diet.

The metabolic profiles of glutamine, Trp, Kyn, Kyn/Trp, glycine, and ornithine were investigated in two or more independent studies. We specifically considered these metabolites to determine whether other potential confounders influenced the findings and our interpretation. We sought to determine if potential confounders, specifically, sex, age, CD4 count, viral load, ART, geographic location, BMI, and diet/fasting state, influenced the metabolic profiles reported in the included studies (Figure 4).

First, we sought to determine whether the reported metabolic profiles are due to a skewed sex distribution in the studies that investigated them. A total of 7/9 studies reported on males for PLHIV, no studies on females, and 2/9 studies reported on mixed‐sex. As most studies reported only males, we were unable to stratify by sex. However, the two studies on mixed‐sex suggests they have dysregulated glutamine, Trp, Kyn, glycine, and ornithine. Therefore, we cannot conclude whether sex has a definite influence on the identified metabolic profiles (Table S3).

Second, we wanted to determine how age influenced the metabolite levels in the studies that were deemed “noteworthy”. Only nine studies reported the mean age of PLHIV. Those nine studies were grouped by age (years): 30–39, 40–49, and 50–59 (Table S4). The studies in the age group 30–39 reported no difference in Kyn and Kyn/Trp metabolite levels in PLHIV compared to HCs, but there were mixed findings for Trp [30, 32, 34, 36]. This age group also had dysregulated ornithine levels in IR PLHIV [35]. The studies in the age group 40–49 reported dysregulation of Kyn, Kyn/Trp, and glycine metabolites [31, 33, 35]. Studies in the age group of 50–59 years old reported dysregulation of all the “noteworthy” metabolites – glutamine, Trp, Kyn, and Kyn/Trp [26, 29]. These findings may suggest increased metabolic alterations with advancing age in PLHIV despite ART; however, interpretation is limited by the small number of studies within each age category (Table S4).

Third, all studies reported CD4 counts higher than 200 cells/mm^3^. Therefore, we were unable to stratify the studies by CD4 count. Notably, despite effective viral control indicated by higher CD4 counts, certain metabolites in PLHIV remain dysregulated compared to HCs.

Fourth, we sought to determine whether viral loads influenced the metabolic profiles reported in the included studies. Most studies (8/10) reported viral load data, all of which showed viral suppression (undetectable/ < 50 copies/mL). Therefore, we were unable to stratify the studies by viral suppression status. As a result, the potential influence of viral load on the metabolic profiles could not be ascertained from this review. However, we noted that all eight studies reporting viral load data indicated viral suppression. The metabolite levels reported in these eight studies remain dysregulated compared with HCs, suggesting a persistent immunometabolic dysregulation, regardless of treatment status and/or viral suppression.

Fifth, all studies included in this systematic review reported that PLHIV were receiving ART. However, only six studies specified the exact ART regimen (Table S5). Given the heterogeneity of reported ART regimens, we classified them by ART class. Of these six studies, five [26, 30, 32, 34, 35] reported the use of nucleoside reverse transcriptase inhibitors (NRTIs) in combination with either integrase strand transfer inhibitors (INSTIs), protease inhibitors (PIs), and non‐nucleoside reverse transcriptase inhibitors (NNRTIs), whereas one study reported the use of PIs alone [80]. The use of ART can induce ART‐associated metabolic changes in PLHIV [38]. However, due to variability in ART drug combinations, we were unable to stratify studies by specific ART classes. As a result, we could not determine if specific ART regimens definitively influence the metabolic levels. Therefore, any ART‐related inferences referred to within this review are speculative. Similarly, ART duration was reported in 10 studies [23, 26, 27, 30, 31, 32, 33, 34, 35, 80]. Most of the metabolites investigated were dysregulated compared with HCs, regardless of long‐term ART use. From the ‘noteworthy’ metabolites, only two studies did not report ART duration [25, 29]. The remaining studies reported ART durations between 6 and 12 months [26, 31, 32] and > 12 months [30, 33, 34, 35]. In the 6–12 months ART duration group, one study reported dysregulation of glutamine, glycine, and ornithine [26] whereas Kyn/Trp showed mixed findings across studies [31, 32]. In the > 12 months ART duration group, two independent studies reported elevated Kyn/Trp [30, 33] whereas one study reported no difference in Kyn/Trp [34] alongside dysregulation of glycine and ornithine [35] (Table S6).

Sixth, we sought to determine if the geographical locations of the reported studies may have influenced the metabolic profiles. Three studies reported on metabolic profiles in the USA [29, 33, 36]. One study reported on Canada (North America) [31], and one study on Sweden (northern Europe) [26]. Three studies reported on metabolic profiles in China (Asia) [30, 32, 34]. Only one study reported on metabolic profiles in South Africa (Africa) [25] and one study did not report on geographical location [35]. To further conceptualise this, we stratified these studies into the Global North and the Global South. Of these studies, four reported on the Global North and five reported on the Global South. Of the four studies from the Global North, all the studies found evidence of metabolic dysregulation in PLHIV. In studies from the Global South, at least 2 of 5 (40%) reported no metabolic dysregulation and 3 of 5 (60%) studies reported metabolic dysregulation in PLHIV compared to HCs (Table S7). Although the number of studies is limited, dysregulation of the commonly investigated metabolic profiles was more commonly reported in studies from the Global North than in those from the Global South. However, further investigation is warranted. However, these findings should be interpreted with caution because of the limited number of studies available for each geographical region, as well as the lack of reported HIV subtype data and inconsistent reporting of ethnicity/race across studies.

Seventh, only one study reported fasting status, with participants fasting overnight prior to sample collection [35], whereas the remaining studies did not report fasting information (Table S8). Similarly, only three studies reported BMI data for participants [26, 31, 35]. Among those studies, BMI did not significantly differ between PLHIV and HCs.

## 4 Discussion

Several key findings emerged from this systematic review. (1) Glutamine, Trp, Kyn, Kyn/Trp, glycine, and ornithine were the most investigated metabolites using targeted metabolomics. (2) A consistent trend was observed across studies in which, compared with HCs, glutamine and glycine levels were lower, while Kyn/Trp and ornithine were higher. These findings warrant further investigation in larger, methodologically standardized studies. (3) Despite higher CD4 counts, viral suppression, and increasing age, altered metabolite profiles were still observed in PLHIV compared with HCs.

A more detailed discussion of the biological significance of these findings will be presented subsequently. Glutamine levels were consistently lower in PLHIV than in HCs [25, 26]. A previous study done in perinatally HIV infected children, however, showed elevated glutamine levels in PLHIV when compared to control groups despite ART status [39], but was shown to decrease after ART commenced. A review done by Mu, Patankar [40] explains the relationship between immune and metabolic dysregulation as a result of HIV infection. HIV infection results in elevated cytokines (interferon gamma in particular). This elevates metabolic rate and tempo, leading to increased glutamine feeding into the tricarboxylic acid (TCA) cycle to meet the increased energy demand. This would necessitate an elevated breakdown of various energy substrates, including muscle protein, leading to elevated serum glutamine levels in untreated adults and children. This is the cause of HIV cachexia, which is a common occurrence in untreated HIV. In the study by Kaur, Oyeyemi [39], a comparative reduction in glutamine was observed in the treated children group compared with the untreated group (although not significantly so), as viral load decreased. In the adult group from the studies in this review [25, 26], who have been on ART for quite some time already, there is a significant reduction. Considering this, the duration of treatment and the subsequent reduction in viral load are the most likely explanations for the observed significant reduction in adults observed in the studies by Svensson Akusjärvi, Krishnan [26] and Sitole, Tugizimana [25], when compared to the smaller reduction in glutamine when comparing treated and untreated children [39]. In a cell culture study specifically investigating T cells, HIV infection significantly elevated intracellular glutamine concentrations compared with uninfected cells [41]. A review by Maciolek, Pasternak [42] on T cells outlines upregulation of glutamine uptake in HIV‐infected cells for energy production via alpha‐ketoglutarate [42, 43] and for lipid, polyamine, and amino acid synthesis [42].

Furthermore, HIV epidemiology, subtype and clinical outcomes differ across geographical regions, potentially influencing underlying metabolic profiles [2, 16]. Given that glutamine dysregulation was reported in studies from both South Africa and Sweden, two distinct geographical locations, the lower glutamine levels may suggest that metabolic dysregulation persists in PLHIV despite these differences and the different diets associated with these populations. Citrulline, a metabolite linked to glutamine metabolism, serves as a biomarker of enterocyte mass and intestinal function [43, 44]. Reduced citrulline levels have been associated with enterocyte loss, impaired gut barrier integrity, and systemic inflammation through inverse associations with inflammatory markers such as C‐reactive protein [45]. Although limited evidence is currently available, these findings may suggest an interaction between amino acid metabolism, intestinal dysfunction, and persistent inflammation in PLHIV.

Considering all the above, however, more studies are needed to provide a clear consensus on glutamine levels in ART‐treated PLHIV, especially across adults and children, and to account for treatment duration and viral load.

Furthermore, as mentioned, Trp, Kyn, and Kyn/Trp were also highlighted in this systematic review as commonly investigated metabolites. These metabolites form part of the Trp‐Kyn pathway [46]. Considering the included studies, reduced Trp and elevated Kyn/Trp were considered “noteworthy” metabolic profiles (criterion 2), whereas Kyn was considered to have mixed findings. Trp, Kyn, and Kyn/Trp share a special relationship as they are the key components in the first step of Trp‐Kyn metabolism. Trp is broken down into Kyn by the IDO enzyme [46] for the eventual synthesis of NAD and ATP, most likely also to meet the energy demands of elevated metabolic rates in HIV infection. To further support this, a systematic review of the Trp‐Kyn pathway in PLHIV found results similar to those in this review, reporting lower Trp levels and a higher Kyn/Trp ratio in ART‐experienced PLHIV compared to HCs [47]. This further supports persistence of altered Trp‐Kyn metabolism despite ART. In a clinical context, this has further relevance, since literature indicates that Trp‐Kyn is dysregulated during HIV infection, and associated with various clinical outcomes in PLHIV, including poor neurological outcomes and even associations with inflammation [46, 47, 48, 49]. Additionally, the Trp‐Kyn metabolic pathway is associated with immune markers, exhibiting an interplay between the Trp‐Kyn pathway and inflammation during HIV infection [50]. IDO is activated by inflammation during HIV infection [51], and in ART‐naïve PLHIV, Kyn/Trp ratio which is an indirect measure of indoleamine 2,3‐dioxygenase (IDO) activity is positively associated with interleukin (IL)‐6, soluble urokinase plasminogen activator receptor (suPAR), and soluble CD (sCD)163 [50]. These immune markers are also associated with CVD [52], atherosclerosis [53], and insulin resistance [54]. These findings may additionally contribute to increased cardiometabolic risk in PLHIV.

Other findings from the studies included in this review provide evidence linking dysregulation of the Trp–Kyn pathway with gut microbial disruption and persistent inflammation in PLHIV despite ART [29, 30, 31, 33]. The Kyn/Trp ratio has been associated with markers of microbial translocation, particularly soluble CD14 (sCD14), which reflects gut barrier dysfunction and systemic immune activation [29, 44, 55]. Although ART reduces viral replication, microbial translocation and chronic inflammation may persist despite treatment [56]. Previous studies have shown positive associations between Kyn and Kyn/Trp with inflammatory markers such as TNF‐α and IL‐6, suggesting that persistent immune activation may continue to stimulate IDO activity and Trp catabolism in PLHIV [31]. Additional evidence suggests that gut epithelial dysfunction, endothelial dysfunction, neutrophil infiltration, epithelial proliferation, and oxidative stress may further contribute to persistent immune‐metabolic dysregulation despite ART [33, 34]. Together, these findings support the biological plausibility that chronic inflammation and persistent gut barrier dysfunction contribute to sustained dysregulation of the Trp–Kyn pathway in ART‐treated PLHIV.

Considering the latter, the changes in glutamine, and considering HIV cachexia, amino acid metabolism is significantly dysregulated in PLHIV, and further investigation of this could provide a clear consensus as to specific amino acid metabolic profiles that are dysregulated during HIV infection.

Ornithine was reported to be higher in ART‐treated PLHIV compared to HCs in two independent studies [26, 35]. Ornithine forms part of the urea cycle (ornithine cycle). Here, toxic ammonia is converted into urea as an excretion mechanism. There are limited studies that have utilised targeted metabolomics to quantify ornithine levels between PLHIV and HCs [26, 35, 57]. In the study by Zhang, Chen [57] PLHIV had a variety of comorbidities, including fatty liver disease. Given that the ornithine cycle occurs in the liver, this may have contributed to the elevated ornithine levels in PLHIV. However, among those studies that investigated ornithine, PLHIV were on ART. This may suggest that ART exposure contributes to altered ornithine metabolism in PLHIV. Specific ART regimens have been reported to have toxic effects/toxicity [58]. These effects may contribute to broader metabolic disturbances. Therefore, the elevated ornithine levels could be due to byproducts of the ART regimens that were administered in the studies by Svensson et al. and Wan et al. Additionally, ornithine also serves as a precursor for polyamines, which play roles in cellular processes such as gene expression, cell proliferation, and cellular stress [59]. In the oral mucosa of PLHIV, disruption of polyamines was seen to impact T cell dysfunction [60]. Collectively, these findings may suggest that ART‐associated hepatobiliary stress and broader metabolic disturbances contribute to altered ornithine metabolism in PLHIV.

Glycine levels were reported to be decreased in two independent studies. Glycine is a precursor for the synthesis of glutathione, which is mainly produced in the liver [61]. The decreased glycine levels reported imply that glutathione synthesis is downregulated. Lowered glutathione is seen during HIV infection and is associated with inflammation in PLHIV [62, 63, 64]. Chronic inflammation is common in ART‐treated PLHIV, and glutathione deficiency is considered a contributing factor, as it causes an increase in cytokine levels [61, 64]. However, glutathione deficiency is combatted by supplementation with glycine, a precursor for the synthesis of glutathione, which has shown improvement in mitochondrial fuel oxidation in the fasted and fed states, insulin sensitivity, muscle strength, body composition, and inflammation [64, 65, 66]. In the studies by Svensson Akusjärvi, Krishnan [26] and Wan, Lam [35] participants were not on any glycine supplementation, which could impair glutathione synthesis.

Holistically, these metabolites participate in interconnected metabolic pathways. Glutamine, ornithine, Trp, Kyn, and glycine are all involved in amino acid metabolism, which is commonly reported to be dysregulated in PLHIV despite ART [67, 68, 69]. Several of these metabolites also converge on related biological pathways. For example, glutamine and ornithine are linked to the urea cycle, where glutamine, through glutaminase (GLS) activity, contributes ammonia for urea cycle metabolism and waste removal [70]. Glutamine additionally contributes to mitochondrial energy metabolism through its role in the TCA cycle. Glycine plays an important role in redox balance and glutathione metabolism, where dysregulation may contribute to oxidative stress [71]. Similarly, Trp, Kyn, and the Kyn/Trp ratio reflect dysregulation of the Trp‐Kyn pathway, which is strongly associated with chronic inflammation and immune activation in PLHIV.

Collectively, these findings suggest that altered metabolite profiles in ART‐treated PLHIV likely reflect broader disruptions in amino acid metabolism, mitochondrial function, oxidative stress pathways, energy metabolism, and immune‐metabolic regulation. These overlapping pathways may further contribute to ART‐associated mitochondrial toxicity and chronic inflammatory processes in PLHIV [72].

From the “noteworthy” metabolites, IDO activity and ornithine were consistently reported to be elevated, whereas glutamine and glycine were reduced in ART‐treated PLHIV compared with HCs (Figure 2). Several potential interventions could be considered to normalise these dysregulated metabolites. Various IDO inhibitors have been proposed as potential strategies to reduce IDO activity in PLHIV [73]. In a study evaluating the combined effect of ART and IDO inhibition in SIV‐infected rhesus macaques, administration of 1‐methyl‐D‐tryptophan inhibited IDO activity and subsequently increased Trp levels after 3 days of treatment [74]. However, Kyn levels did not decrease throughout the treatment period [74].

Potential interventions may also include glutamine and glycine supplementation to restore their reduced levels (Cruzat et al., 2018; Djordjevic et al., 2026; Ramos‐Jiménez et al., 2024). Glycine supplementation combined with N‐acetylcysteine (GlyNAC) has been investigated in PLHIV and was associated with improvements in glutathione‐related defects of ageing, including oxidative stress, mitochondrial dysfunction, inflammation, endothelial dysfunction, insulin resistance, strength, and cognition [75]. Additionally, alanyl‐glutamine supplementation was associated with improved intestinal absorption in PLHIV [76].

Conversely, elevated ornithine levels may potentially be moderated through dietary regulation of amino acid intake, particularly arginine‐ and ornithine‐rich foods or supplements. L‐arginine can be metabolised through two pathways: by nitric oxide synthase to produce nitric oxide, or by arginase to produce ornithine and urea. Increased arginase activity may therefore contribute to elevated ornithine levels, as observed in the studies included in this review, while also impairing immune function. Inhibitors such as Nω‐hydroxy‐nor‐L‐arginine (nor‐NOHA) redirect L‐arginine metabolism away from urea and ornithine production in HIV/TB co‐infected models [77]. Although these intervention strategies appear promising, they remain underexplored and warrant further investigation, particularly in ART‐treated PLHIV with persistent dysregulation of glutamine, glycine, ornithine, and Kyn/Trp despite viral suppression.

Most ‘noteworthy’ metabolites were reported in plasma. However, one study reported glutamine levels in serum [25]. Both plasma and serum are commonly used in metabolomics investigations, although plasma is generally considered more reproducible, whereas serum may contain higher metabolite concentrations. The only metabolite that could be compared across both matrices was glutamine [25, 26]. Notably, glutamine levels were consistently lower in PLHIV compared with HCs in both the plasma‐ and serum‐based studies (Figure 3). Although broader matrix‐stratified comparisons were not feasible due to the limited number of serum‐based studies, these findings suggest consistency in the direction of glutamine dysregulation across sample types. Nevertheless, future targeted metabolomics studies should standardise and clearly report biological matrices to improve comparability across studies.

Although ART regimens were heterogeneous across the included studies, limiting meaningful comparisons regarding regimen‐specific metabolic effects, ART itself remains an important contributor to metabolic alterations in PLHIV. While ART effectively suppresses HIV replication, it may also contribute to metabolic complications, including diabetes, lipodystrophy, obesity, CVD, and metabolic syndrome [38, 78, 79]. Previous studies have reported ART‐associated alterations in triglycerides, total cholesterol, LDL‐C, glucose metabolism, insulin resistance, liver enzymes (AST and ALT), and bilirubin levels [38, 78].

Specific ART classes may differentially influence metabolic pathways. Protease inhibitor (PI)‐based regimens are strongly associated with dyslipidemia, central obesity, lipodystrophy, elevated triglycerides, and insulin resistance [82, 83]. In contrast, integrase strand transfer inhibitors (INSTIs), although generally associated with improved lipid profiles relative to PIs and NNRTIs [84], have increasingly been linked to weight gain, hyperglycemia, hepatic steatosis, and new‐onset diabetes mellitus [85, 86, 87]. Furthermore, Ursenbach et al. (2020) reported differing incidences of diabetes across ART classes, although age and BMI appeared to be stronger predictors than ART regimen alone [85]. Collectively, these findings suggest that ART‐associated effects may contribute to persistent metabolic alterations despite viral suppression. However, due to inconsistent reporting of ART regimens and combinations across the included studies, definitive conclusions regarding regimen‐specific metabolite changes could not be made in this review.

Differences in metabolite profiles between studies from the Global North and Global South may reflect broader epidemiological and non‐viral influences rather than HIV subtype variation alone. Factors such as diet, socioeconomic status, healthcare access, BMI, fasting status, age, sex, and cohort composition may substantially influence metabolite levels in PLHIV. However, these variables were inconsistently reported across studies, limiting further stratified analyses. Although preliminary evidence suggests that HIV subtype diversity may contribute to metabolic variation, this remains speculative because subtype information was not consistently reported in the included studies. Future targeted metabolomics studies should therefore incorporate standardized reporting of HIV subtype, BMI, fasting status, dietary information, and other relevant covariates to improve interpretation and comparability across regions.

ART, particularly PIs, is associated with hepatotoxicity in PLHIV, including elevations in alanine aminotransferase (ALT) and aspartate aminotransferase (AST), likely due to hepatic metabolism of these agents [88, 89]. Given that the ornithine (urea) cycle primarily occurs in the liver, hepatobiliary alterations associated with ART may contribute to dysregulation of ornithine metabolism. Notably, the studies reporting elevated ornithine levels in this review involved participants receiving PI‐based ART regimens [26, 35], although direct causal associations cannot be established from the currently available evidence.

In addition to potential ART‐associated hepatotoxicity, disturbances in amino acid metabolism, chronic inflammation, mitochondrial dysfunction, and metabolic syndrome‐related processes may also contribute to altered ornithine levels in PLHIV. Previous studies have reported disruptions in urea cycle metabolites, including arginine and urea, during HIV infection [67]. Reduced arginine availability has additionally been associated with inflammation and endothelial dysfunction [90], which may further influence urea cycle homoeostasis and amino acid metabolism. Collectively, these findings suggest that elevated ornithine levels in ART‐treated PLHIV are likely multifactorial and may reflect overlapping effects of ART exposure, hepatobiliary stress, chronic inflammation, and broader metabolic dysregulation rather than a single isolated mechanism.

Dysregulated metabolites were observed in both the 6–12 months and > 12 months ART duration groups, suggesting that metabolic dysregulation may persist despite prolonged ART exposure. However, these findings remain limited by the small number of available studies within each ART‐duration category. Mixed findings relating to Kyn/Trp may reflect differences in immune activation prior to ART initiation, timing of treatment initiation, viral suppression, and baseline CD4 counts [91]. Previous evidence suggests that ART may reduce, but not completely normalize, IDO activity and associated Trp–Kyn pathway dysregulation in PLHIV [32]. Persistent chronic inflammation despite virological suppression may therefore continue to contribute to altered amino acid metabolism in ART‐treated PLHIV.

Although most studies included in this review were conducted before long‐acting ART regimens were approved in 2021, future studies should consider including participants receiving long‐acting ART. This may provide valuable insight into how sustained, steady‐state drug delivery influences metabolite profiles over time compared with conventional daily oral ART regimens. Long‐acting ART has emerged as a promising therapeutic strategy and is associated with improved adherence, stable drug concentrations, maintained virological suppression, fewer drug‐drug interactions, improved quality of life, greater treatment discretion, and potentially fewer treatment‐related side effects [92]. Although targeted metabolomics studies investigating long‐acting ART remain limited, current evidence suggests no significant changes in lipid profiles, glucose levels, or weight associated with these regimens [92]. Future targeted metabolomics studies investigating long‐acting ART may therefore provide important insight into the long‐term metabolic consequences of sustained ART exposure in PLHIV.

There was considerable heterogeneity in the analytical instrumentation and metabolomics methodologies used across the included studies. Different analytical platforms possess varying sensitivity, specificity, dynamic range, and quantitative capabilities, which may influence metabolite detection and quantification [93]. Variability was additionally observed in the use of reference standards, internal standards, protein removal procedures, extraction techniques, and calibration strategies [93]. Such methodological differences may contribute to variability in reported metabolite concentrations across studies [94].

Most studies used methanol‐based protein precipitation; however, alternative protein removal and extraction approaches were also reported. These methodological differences may influence metabolite recovery and stability due to differing chemical interactions and extraction efficiencies [95, 96]. Similarly, studies inconsistently reported quality control procedures, batch correction, limits of detection (LOD), and limits of quantification (LOQ), which further limits direct quantitative comparison between studies. Batch effects, defined as systematic non‐biological variation introduced during sample processing or analytical acquisition, may influence metabolomics datasets if not appropriately assessed or corrected [97].

Differences in LOD and LOQ likely reflect variation in analytical platform sensitivity and calibration procedures [98]. Furthermore, the use of different reference standards and calibration strategies across studies may contribute additional variability in metabolite quantification [99]. Therefore, caution should be exercised when comparing absolute metabolite concentrations across studies using different analytical methodologies and platforms.

Considering that most of the PLHIV in the included studies were on ART, it is not surprising that most studies had comparatively high CD4 counts and low viral load [100]. Despite this, altered metabolite profiles were still observed in PLHIV compared with HCs. This further supports the notion that chronic inflammation in PLHIV persists despite ART, resulting in an elevated Kyn/Trp ratio [50, 51, 101]. However, these findings should be interpreted with caution. There are important limitations to consider relating to the heterogeneity in study design, analytical platforms, and geographical settings, together with inconsistent reporting of key confounders, that is, diet, fasting status, and clinical variables, which may have influenced cross‐study comparability of the included studies. Additional study limitations are discussed in Section 5 (Limitations).

## 5 Limitations

First, due to the heterogeneity of study designs, a meta‐analysis was not conducted. The included studies reported different units of measurement for metabolite quantification, some did not report *p*‐values, and some reported inconsistent units and effect sizes. Additionally, differences in methodology (sample preparation, internal standards, columns, etc.) and the sensitivity of the metabolomics analytical platforms in the included studies further prevented us from conducting a meta‐analysis. Three studies included in this review reported effect sizes without accompanying *p*‐values when comparing metabolic profiles between PLHIV and HCs. These studies were included because of the limited number of studies available on this topic. While effect sizes provide valuable information regarding the magnitude of observed differences between groups, the absence of *p*‐values limits statistical inference and reduces confidence when comparing findings across studies. This inconsistency in reporting statistical outcomes should therefore be taken into account when interpreting the overall findings of this review.

Second, due to variability in reporting, we did not comment on the metabolomics methodologies used in the included studies. Therefore, the findings where more than one metabolite is reported should be interpreted with caution. Third, cART regimens were inconsistently reported across studies, making it difficult to attribute findings to specific regimens, as they may also influence metabolic dysregulation in PLHIV [38]. Thus, ART‐related inferences within this review are speculative. Fourth, almost all studies reported plasma metabolic profiles, and only one reported serum metabolic profiles. This may influence the metabolic profiles reported. Plasma and serum are both suitable for metabolomics, with plasma providing better reproducibility than serum [102]. However, serum offers higher sensitivity and higher metabolite concentrations than plasma, making it the most favourable choice for metabolomics research, particularly for biomarker detection [102].

Fifth, sex may also influence differences in the identification of metabolic profiles [18]. However, almost all studies reported more male PLHIV, thereby not allowing stratification by sex. However, given the limited studies reported in this review, it cannot be determined with certainty that sex influences the reported profiles, as these studies also did not compare metabolite differences between males and females. Thus, further studies on this topic should be done comparing male and female PLHIV to assess whether sex differences may have played a role in the metabolic profiles reported in the included studies. Sixth, the studies in this review were done in different geographical locations. Different HIV subtypes exist in different regions of the world that show differences in clinical outcomes. Ideally, analyses would be conducted by HIV subtype but studies do not report on this, whereas limited studies per region does not allow for meaningful comparisons. Future studies with larger, more diverse datasets should aim to address this in addition to reporting on HIV subtypes to allow us to make such stratifications. Seventh, based on the set quality assessment criteria, most studies were rated as high quality. However, because there was no set standard method, which created heterogeneity in the methods, and some studies did not report the ART regimens used, studies with lower rankings may have been classified as higher quality. Eighth, we included three studies that reported only effect sizes, not *p*‐values, to compare metabolic profiles between PLHIV and HCs [23, 25, 80]. Although these studies did not report statistical significance for the difference between the PLHIV and HCs, we included them because of the limited number of studies on this topic.

Ninth, not all studies reported on the diets or if participants were in a fasting state when sample was collected. Thus, reducing interpretation of diet on the metabolite levels. Population background modifies associations between metabolomic profiles and clinical outcomes [103, 104]. There are several factors, including BMI, fasting status, diet, and other clinical covariates, that may influence the metabolite profiles reported across the included studies. However, these variables were inconsistently reported, limiting further stratified analyses and interpretation of their contribution to the observed metabolite differences. Therefore, findings should be interpreted cautiously. Future studies should incorporate detailed reporting of HIV subtype, ethnicity/race, geographical location, BMI, diet, and fasting status to improve interpretation of population‐specific influences on metabolic profiles in ART‐treated PLHIV.

Tenth, we acknowledge that some included studies primarily investigated metabolomic profiles in populations with comorbidities or substance use. Such studies were only included if they contained a subgroup comparison between ART‐treated PLHIV (with no co‐infections) and HIV‐uninfected controls without the relevant comorbidity or exposure of interest. In these cases, only data relevant to the PLHIV versus HIV‐uninfected HCs comparison were extracted and included in this review. This approach was applied, for example, in the study by Kostadinova et al. (2016) [24].

Lastly, various metabolomics analytical platforms were used and subsequently, different sample preparations were utilised in these studies. Different analytical platforms have advantages and disadvantages relative to others (e.g., reproducibility, sample destruction, sensitivity) [105]. ^1^H‐NMR offers better reproducibility and non‐destructive sample preparation but lower sensitivity than MS‐based metabolomics coupled to LC or GC [106]. Although MS coupled to LC or GC offers better sensitivity, it often requires destructive sample preparation, such as ionisation and derivatisation, to achieve this sensitivity and may lead to metabolite losses [107]. The GC is more suited for volatile metabolites and typically requires derivatisation [105]. Quantification of the metabolites typically requires reference standards of known concentration [107, 108]. However, there is a lack of standard reference material for many metabolites [109]. Therefore, this further contributes to the variability when comparing metabolite levels across different studies. Therefore, due to variability in sample preparation across the included studies, we have not compared how these procedures may have influenced the quantification of the metabolites of interest. However, we do note that these may have contributed to the findings of the included studies and ultimately the interpretation of the conclusions of this review. Therefore, findings from these three studies should be interpreted with caution when comparing metabolic profiles between PLHIV and HCs.

## 6 Conclusion

In this systematic review, we compile evidence from targeted metabolomics studies of ART‐experienced PLHIV compared with HCs. We highlight that reductions in glutamine and glycine, accompanied by elevations in Kyn/Trp and ornithine, and variations in Kyn and Trp, are among the most investigated metabolites, and we elaborate on their biological significance and the mechanisms at play in ART‐treated PLHIV. We also observe that metabolic dysregulation persists despite high CD4 counts and viral suppression. This systematic review serves as a basis for identifying current consensus, limitations, and gaps in current HIV research, guiding future investigations on the topic of HIV infection and PLHIV in the modern ART‐era.

## Author Contributions

LKA and MEW conceived the study, developed the search strategy, and conducted the database searches and screening process. LKA performed the data extraction and prepared the first draft of the manuscript. EJVV, DL, and SM provided intellectual input, methodological guidance, and supervision throughout the study. All authors contributed to the interpretation of the findings, critically reviewed and revised the manuscript, and approved the final version for publication.

## Funding

MEW was funded by the South African National Research Foundation (NRF; TTK22031652) and Poliomyelitis Research Foundation (PRF; 23/84). For LKA, this work is based on the research supported in part by the National Research Foundation of South Africa (Reference Number: AHPMDS250328307160) and PRF (grant number: 25/54).

## Ethics Statement

Ethical approval for this study was obtained from the Health Research Ethics Committee of North‐West University (NWU‐HEREC; NWU‐00063‐26‐A1).

## Consent

The authors have nothing to report.

## Conflicts of Interest

The authors declare no conflicts of interest.

## Supporting information


Supporting Information S1

Supporting Information S2

**Table S1:** Quality assessment of the included studies.

**Table S2:** Metabolomics experimental information.

**Table S3:** Stratification of the included studies according to sex.

**Table S4:** Stratification of the included studies according to age of PLHIV.

**Table S5:** ART information and HIV infection duration of the included studies.

**Table S6:** Stratification of the included studies according to ART duration.

**Table S7:** Stratification of the included studies according to the Global North and the Global South.

**Table S8:** Studies reporting on factors that could influence “noteworthy” metabolites.

## Data Availability

The data that support the findings of this study are available from the corresponding author upon reasonable request.
