# Supplementary material for: Dysregulated Metabolism in People Living With HIV in the Modern ART‐Era: A Systematic Review of Targeted Metabolomics Studies
Source: Rev Med Virol. 2026 Jul 6;36(4):e70179. doi: 10.1002/rmv.70179 (PMC13335820; doi:10.1002/rmv.70179)
Supplement: Supplementary file 1 — Supporting Information S1 [file RMV-36-e70179-s004.docx]

**Supplementary File 1**

**27th of August 2025**

**Pubmed: 1369 articles**

(Human immunodeficiency virus [tw] OR Human immunodeficiency virus [mh]) AND (liquid chromatography [tw] OR chromatography, liquid [mh] OR Gas chromatography [tw] OR Chromatography, Gas [mh] OR nuclear magnetic resonance [tw] OR magnetic resonance spectroscopy [tw] OR Magnetic Resonance Spectroscopy [mh] OR mass spectrometry [mh] OR tandem mass spectrometry [mh] OR targeted metabolomics [tw] OR metabolomics [mh]) AND (blood [tw] OR blood [mh] OR serum [tw] OR plasma [tw])

**27th of August 2025**

**Scopus: 707 articles**

(hiv OR "human immunodeficiency virus" OR aids OR "acquired immunodeficiency syndrome" ) AND ( "targeted metabolomics" ) AND ( blood OR serum OR plasma )

**27th of August 2025**

**Web of Science: 941 articles**

TS=(human immunodeficiency virus) AND TS=(liquid chromatography OR gas chromatography OR magnetic resonance spectroscopy OR mass spectrometry OR tandem mass spectrometry OR targeted metabolomics OR metabolomics) AND TS=(blood OR serum OR plasma)
