# Supplementary material for: Dysregulated Metabolism in People Living With HIV in the Modern ART‐Era: A Systematic Review of Targeted Metabolomics Studies
Source: Rev Med Virol. 2026 Jul 6;36(4):e70179. doi: 10.1002/rmv.70179 (PMC13335820; doi:10.1002/rmv.70179)
Supplement: Supplementary file 2 — Supporting Information S2 [file RMV-36-e70179-s001.docx]

**Supplementary file 2: An extensive list of all the metabolites that are significantly different in PLHIV and HCs.**

**The following metabolites were different between PLHIV INRs and HCs (p < 0.05):**

Taurine

L-Valine

N-Formyl-L-methionine

L-Leucine

L-Lysine

Glycylhydroxyproline

gamma-Glutamylalanine

N6-Acetyl-L-lysine

L-Tyrosine

Histidylthreonine

N2-gamma-Glutamylglutamine

gamma-Glutamylmethionine

L-Tryptophan

Beta-Citryl-L-glutamic acid

Cysteineglutathione disulfide

Imazamethabenz

Isomer 1 of imazamethabenz

Isomer 2 of imazamethabenz

2-[2-(1,3-Dioxo-1,3-dihydro-isoindol-2-yl)-acetylamino]-4-methyl-pentanoic acid

Indolebutyroyl aspartic acid

Pro Ser Ser Val

Dityrosine

Thr Leu Phe Tyr

3-Hydroxybenzoic acid

2,4-dihydroxy-3-methoxybenzoic acid

4-methoxygentisic acid

Chenodeoxycholic acid

Ursodeoxycholic acid

Cholic acid

Chenodeoxyglycocholic acid

Bilirubin

Glyceric acid

Threonic Acid

Arabinonic acid

Glucuronic acid/Galaturonic acid

Gluconic acid

N-Acetylmuramate

N-Acetylneuraminic acid

p-Cresol glucuronide

7-Hydroxyterpineol 8-glucoside

3-(2,3,4-trimethoxyphenyl)propanoic acid

2-Ethyl-2-Hydroxybutyric acid

Hydroxyisocaproic acid

Mevalonic acid

2-Octenedioic acid

3-Hydroxyoctanoic acid

Decadienedioic acid

(8Z)-dec-8-enoic acid

3-Oxododecanoic acid

Goshuyic acid

5-Tetradecenoic acid

12-Methyltridecanoic acid

7Z,10Z-Hexadecadienoic acid

Palmitoleic acid

9Z-Heptadecenoic acid

Alpha-Linolenic acid

Linoleic acid

Oleic acid

9,12,13-TriHOME

Arachidonic acid

CPA(18:2(9Z,12Z)/0:0)

FFA C22:5

2-Hydroxybutyric acid

2,4-Dihydroxybutanoic acid

3-Hydroxydodecanoic acid

Urocanic acid

5-Hydroxyindoleacetic acid

Methyl pyruvate*

Oxoglutaric acid*

3-Oxodecanoic acid

Inosinic acid

Ribothymidine

AMP

2-(2,6-Dioxo-1,2,3,6-tetrahydropyrimidin-4-yl)ethanesulfonicacid

p-Cresol sulfate

Dihydrocaffeic acid 3-sulfate

O-Phosphoethanolamine

2-Methoxyhydroquinone

[3-(4-methoxyphenyl)propoxy]sulfonic acid

Phenylgalactoside

23S,25,26-Trihydroxyvitamin D3

1a-1-hydroxy-2,4(18),11(13)-eudesmatrien-12-Oate

Dihydrouracil

Deoxyeritadenine

16alpha-Hydroxy DHEA 3-sulfate

Androsterone sulfate

Stanolone glucuronate

Fusicoccin H

L-Acetylcarnitine

Butyrylcarnitine

Isobutyrylcarnitine

(R)-3-hydroxybutyrylcarnitine

3-Methylglutarylcarnitine

3-hydroxyoctanoyl carnitine

isomer of 3-hydroxyoctanoyl carnitine

3-hydroxydecanoyl carnitine

3-hydroxydodecanoyl carnitine

3, 5-Tetradecadiencarnitine

cis-5-Tetradecenoylcarnitine

Tetradecanoylcarnitine

(2E)-Hexadecenoyl-carnitine

L-Palmitoylcarnitine

Gamma-linolenyl carnitine

Linoelaidyl carnitine

Oleoylcarnitine

Arachidonoylcarnitine

N-(1-Deoxy-1-fructosyl)leucine

N-(1-Deoxy-1-fructosyl)isoleucine

Glycine

Creatinine

L-Methionine

Methionine sulfone

L-Proline

trans-S-(1-Propenyl)-L-cysteine

L-Arginine

1-Methylhistidine

Isovalerylglycine

1-Carboxy-L-prolylglycine

N-gamma-Glutamylglutamine

Valylhydroxyproline

Tyrosyl-Phenylalanine

Tryptophyl-Tryptophan

Isomer of Tryptophyl-Tryptophan

Arg Trp Cys His

4-Ethoxy-3-anisaldehyde

4-(3-Methyl-5-oxo-4,5-dihydro-1H-pyrazol-1-yl)benzoic acid

Dillapiol

2-Aminobenzoic acid

Glucosamine

Netilmicin

Acetylcholine

Oleamide

Glycerophosphocholine

sn2 LysoPC(18:2)

Trimethylamine N-oxide

1-Pyrroline

Pyrrolidine

2-Methylthiazolidine

L-Formylkynurenine

Sphinganine

2-Hydroxyadenine

Thymine

2-Acetyl-D1-piperideine

Hydroxycotinine

Phosphorylcholine

O-Methylcorypalline

**The following metabolites were different between the PLHIV IRs and HCs:**

Taurine

gamma-Aminobutyric acid

L-Valine

Pyroglutamic acid

L-Glutamic acid

N-Formyl-L-methionine

D-xylo-Form

2-Methylbutyrylglycine

4-Hydroxystachydrine

Aspartyl-Threonine

Phenylacetylglycine

3-Methoxytyrosine

Histidylthreonine

N2-gamma-Glutamylglutamine

gamma-Glutamylmethionine

L-Tryptophan

5-Hydroxy-L-tryptophan

Beta-Citryl-L-glutamic acid

Tyrosyl-Alanine

Hypoglycin B

Isomer of hypoglycin B

Phenylalanylaspartic acid

Cysteineglutathione disulfide

Imazamethabenz

Isomer 1 of imazamethabenz

Isomer 2 of imazamethabenz

2-[2-(1,3-Dioxo-1,3-dihydro-isoindol-2-yl)-acetylamino]-4-methyl-pentanoic acid

Indolebutyroyl aspartic acid

Pro Ser Ser Val

Dityrosine

Thr Leu Phe Tyr

Benzenebutanoic acid

3-Hydroxybenzoic acid

4-methoxygentisic acid

2-(pyridin-2-ylamino)benzoic acid

3-Methyl-1,4-dioxo-1,4-dihydronaphthalene-2-carboxylic acid

Chenodeoxycholic acid

Ursodeoxycholic acid

Cholic acid

Chenodeoxyglycocholic acid

Glycochenodeoxycholic acid

Bilirubin

Glyceric acid

Arabinonic acid

Glucuronic acid/Galaturonic acid

5,6-Dihydrouridine

N-Acetylmuramate

7-Hydroxyterpineol 8-glucoside

Citric acid

2-Ethyl-2-Hydroxybutyric acid

Mevalonic acid

3-Hydroxyoctanoic acid

Decadienedioic acid

3-Oxododecanoic acid

Goshuyic acid

5-Tetradecenoic acid

2-methyl-tridecanedioic acid

12-Methyltridecanoic acid

7Z,10Z-Hexadecadienoic acid

Palmitoleic acid

9Z-Heptadecenoic acid

Alpha-Linolenic acid

Linoleic acid

Oleic acid

9,12,13-TriHOME

Arachidonic acid

CPA(18:2(9Z,12Z)/0:0)

L-Lactic acid

2-Deoxypentonic acid

3-Hydroxydodecanoic acid

Urocanic acid

1H-Indole-3-carboxaldehyde

2-Ketobutyric acid*

Methyl pyruvate*

3'-UMP/5'-UMP

Inosine

Ribothymidine

2-Hydroxy-3-(sulfooxy)benzoate

O-Phosphoethanolamine

Allantoin

2-Methoxyhydroquinone

Phenylgalactoside

23S,25,26-Trihydroxyvitamin D3

16alpha-Hydroxy DHEA 3-sulfate

Androsterone sulfate

Epiandrosterone Sulfate

L-Acetylcarnitine

Isobutyrylcarnitine

Isovalerylcarnitine

2-Octenoylcarnitine

3-hydroxyoctanoyl carnitine

isomer of 3-hydroxyoctanoyl carnitine

3-hydroxydecanoyl carnitine

4,8 Dimethylnonanoyl carnitine

3-Hydroxy-cis-5-tetradecenoylcarnitine

Tetradecanoylcarnitine

(2E)-Hexadecenoyl-carnitine

L-Palmitoylcarnitine

Gamma-linolenyl carnitine

Linoelaidyl carnitine

Oleoylcarnitine

Arachidonoylcarnitine

Betaine

L-Methionine

Methionine sulfone

Ornithine

L-Arginine

1-Carboxy-L-prolylglycine

Asymmetric dimethylarginine

N-gamma-Glutamylglutamine

Glutamylphenylalanine

Tyrosyl-Phenylalanine

Tryptophyl-Tryptophan

Isomer of Tryptophyl-Tryptophan

Arg Trp Cys His

4-(3-Methyl-5-oxo-4,5-dihydro-1H-pyrazol-1-yl)benzoic acid

3-Amino-9-ethylcarbazole

Dihydroactinidiolide

2-Aminobenzoic acid

Nitroxazepine

Acetylcholine

Glycerophosphocholine

sn2 LysoPC(14:0)

sn1 LysoPC(14:0)

sn2 LysoPC(16:1)

sn1 LysoPC(16:1)

sn2 LysoPC(18:2)

sn2 LysoPC(20:3)

Metabolites

sn2 LysoPE(22:6)

sn1 LysoPE(22:6)

1-Methylinosine

Pyrrolidine

2-Methylthiazolidine

L-Formylkynurenine

Sphinganine

2-Hydroxyadenine

Hypoxanthine

Thymine

1-Methylguanine

Hydroxycotinine

5'-Hydroxycotinine

Phosphorylcholine

O-Methylcorypalline
