# Supplementary material for: Dysregulated Metabolism in People Living With HIV in the Modern ART‐Era: A Systematic Review of Targeted Metabolomics Studies
Source: Rev Med Virol. 2026 Jul 6;36(4):e70179. doi: 10.1002/rmv.70179 (PMC13335820; doi:10.1002/rmv.70179)
Supplement: Supplementary file 3 — Table S1: Quality assessment of the included studies. [file RMV-36-e70179-s007.docx]

**Supplementary Table 1:** Quality assessment of the included studies

| **Quality Assessment LA** | | | | | |
| --- | --- | --- | --- | --- | --- |
| Reference | Question 1 | Question 2 | Question 3 | Summed rating | Overall comment |
| [1] | 2 | 2 | 1 | 5 | High |
| [2] | 2 | 2 | 1 | 5 | High |
| [3] | 2 | 2 | 2 | 6 | High |
| [4] | 2 | 2 | 2 | 6 | High |
| [5] | 1 | 2 | 1 | 4 | Intermediate |
| [6] | 1 | 2 | 1 | 4 | Intermediate |
| [7] | 1 | 1 | 1 | 3 | Intermediate |
| [8] | 2 | 1 | 2 | 5 | High |
| [9] | 2 | 1 | 1 | 4 | Intermediate |
| [10] | 2 | 1 | 2 | 5 | High |
| [11] | 2 | 1 | 1 | 4 | Intermediate |
| [12] | 2 | 1 | 2 | 5 | High |
| [13] | 2 | 1 | 1 | 4 | Intermediate |
| [14] | 1 | 1 | 2 | 4 | Intermediate |
| [15] | 1 | 1 | 2 | 4 | Intermediate |
| **Quality Assessment MW** | | | | | |
| Reference | Question 1 | Question 2 | Question 3 | Summed rating | Overall comment |
| [1] | 2 | 1 | 1 | 4 | Intermediate |
| [2] | 2 | 2 | 1 | 5 | High |
| [3] | 2 | 2 | 1 | 5 | High |
| [4] | 2 | 2 | 1 | 5 | High |
| [5] | 2 | 2 | 0 | 4 | Intermediate |
| [6] | 1 | 2 | 1 | 5 | Intermediate |
| [7] | 1 | 1 | 1 | 3 | Intermediate |
| [8] | 2 | 2 | 1 | 5 | High |
| [9] | 1 | 1 | 1 | 3 | Intermediate |
| [10] | 2 | 1 | 2 | 5 | High |
| [11] | 1 | 2 | 1 | 4 | Intermediate |
| [12] | 1 | 1 | 1 | 3 | Intermediate |
| [13] | 2 | 1 | 2 | 5 | High |
| [14] | 1 | 1 | 2 | 4 | Intermediate |
| [15] | 1 | 1 | 2 | 4 | Intermediate |

**References**

1. Gebremicael G, Alemayehu M, Sileshi M, et al. The serum concentration of vitamin B(12) as a biomarker of therapeutic response in tuberculosis patients with and without human immunodeficiency virus (HIV) infection. *Int J Gen Med* 2019; 12: 353-361. DOI: 10.2147/ijgm.S218799

2. Kostadinova L, Shive CL, Judge C, et al. During Hepatitis C Virus (HCV) Infection and HCV-HIV Coinfection, an Elevated Plasma Level of Autotaxin Is Associated With Lysophosphatidic Acid and Markers of Immune Activation That Normalize During Interferon-Free HCV Therapy. *J Infect Dis* 2016; 214: 1438-1448. DOI: 10.1093/infdis/jiw372

3. Sitole LJ, Tugizimana F, Meyer D. Multi-platform metabonomics unravel amino acids as markers of HIV/combination antiretroviral therapy-induced oxidative stress. *J Pharm Biomed Anal* 2019; 176: 112796. DOI: 10.1016/j.jpba.2019.112796

4. Svensson Akusjärvi S, Krishnan S, Ambikan AT, et al. Role of myeloid cells in system-level immunometabolic dysregulation during prolonged successful HIV-1 treatment. *Aids* 2023; 37: 1023-1033. DOI: 10.1097/qad.0000000000003512

5. van der Ven AJ, Blom HJ, Peters W, et al. Glutathione homeostasis is disturbed in CD4-positive lymphocytes of HIV-seropositive individuals. *Eur J Clin Invest* 1998; 28: 187-193. DOI: 10.1046/j.1365-2362.1998.00267.x

6. McRae M, Rezk NL, Bridges AS, et al. Plasma bile acid concentrations in patients with human immunodeficiency virus infection receiving protease inhibitor therapy: possible implications for hepatotoxicity. *Pharmacotherapy* 2010; 30: 17-24. DOI: 10.1592/phco.30.1.17

7. Neves FF, Vannucchi H, Jordão AA, Jr., Figueiredo JF. Recommended dose for repair of serum vitamin A levels in patients with HIV infection/AIDS may be insufficient because of high urinary losses. *Nutrition* 2006; 22: 483-489. DOI: 10.1016/j.nut.2005.11.008

8. Baer SL, Colombo RE, Johnson MH, et al. Indoleamine 2,3 dioxygenase, age, and immune activation in people living with HIV. *J Investig Med* 2021; 69: 1238-1244. DOI: 10.1136/jim-2021-001794

9. Chen J, Shao J, Cai R, et al. Anti-retroviral therapy decreases but does not normalize indoleamine 2,3-dioxygenase activity in HIV-infected patients. *PLoS One* 2014; 9: e100446. DOI: 10.1371/journal.pone.0100446

10. Chen J, Xun J, Yang J, et al. Plasma Indoleamine 2,3-Dioxygenase Activity Is Associated With the Size of the Human Immunodeficiency Virus Reservoir in Patients Receiving Antiretroviral Therapy. *Clin Infect Dis* 2019; 68: 1274-1281. DOI: 10.1093/cid/ciy676

11. Jenabian MA, Patel M, Kema I, et al. Distinct tryptophan catabolism and Th17/Treg balance in HIV progressors and elite controllers. *PLoS One* 2013; 8: e78146. DOI: 10.1371/journal.pone.0078146

12. Somsouk M, Estes JD, Deleage C, et al. Gut epithelial barrier and systemic inflammation during chronic HIV infection. *Aids* 2015; 29: 43-51. DOI: 10.1097/qad.0000000000000511

13. Yang J, Cai R, Xun J, et al. Elevated indoleamine 2,3-dioxygenase activity is associated with endothelial dysfunction in people living with HIV and ROS production in human aortic endothelial cells in vitro. *Drug Discov Ther* 2023; 17: 312-319. DOI: 10.5582/ddt.2023.01069

14. Wan LY, Lam SM, Huang HH, et al. Multi-omics dissection of metabolic dysregulation associated with immune recovery in people living with HIV-1. *J Transl Med* 2025; 23: 143. DOI: 10.1186/s12967-025-06168-0

15. Cherenack EM, Larson ME, Murray K, et al. Stimulant Use, HIV, and Plasma Metabolites Among Men. *Journal of Neuroimmune Pharmacology* 2025; 20: 68. DOI: 10.1007/s11481-025-10223-4
