# Supplementary material for: Dysregulated Metabolism in People Living With HIV in the Modern ART‐Era: A Systematic Review of Targeted Metabolomics Studies
Source: Rev Med Virol. 2026 Jul 6;36(4):e70179. doi: 10.1002/rmv.70179 (PMC13335820; doi:10.1002/rmv.70179)
Supplement: Supplementary file 4 — Table S2: Metabolomics experimental information. [file RMV-36-e70179-s005.docx]

**Supplementary Table 2:** Metabolomics experimental information

| **Reference** | **Reference standard** | **Protein removal** | **Extraction** | **Quality control** | **Batch correction** | **Limit of detection and/or quantification** |
| --- | --- | --- | --- | --- | --- | --- |
| [1] | Internal standard:   - Retinyl acetate | Ethanol | Hexane | N/D | N/D | - LOD: N/D - LOQ: N/D |
| [2] | Internal standard:   - LPA 17:0 | Methanol | N/D | N/D | N/D | - LOD: 0.05 uM - LOQ: N/D |
| [3]* | Calibration standards:   - Alanine - 2-amino-butyric-XXXX - Asparagine - Aspartic acid - Cystathionine - Cystine - Glutamic acid - Glutamine - Glycine - Histidine - Isoleucine - Leucine - Lysine - Methionine - Ornithine - Phenylalanine - Proline - Serine - Threonine - Tryptophan - Tyrosine - Valine | 10kDa centrifugal filter | Solid-phase extraction followed by derivatisation followed by liquid/liquid extraction | - External quality controls (commercial lyophilized human serum samples spiked with concentrations of amino acids) - Calibration standards (commercial standard mixtures of amino acid as part of the EZ:faast™ analysis kit) | No batch effects | - Reports on reliability of the instrument in detecting all calibration standards |
| [4]* | LC: Used internal standards but they are N/D  GC: N/D | Methanol | N/D | - Pooled quality control - Blank controls - Cocktail quality control standards | Only one batch. Thus, no batch effect. | - Determine instrument reliability using internal standards added to each sample |
| [5]* | Not well described | Not well described | Not well described | Not well described | Not well described | Not well described |
| [6] | Internal standards:   - N-1 naphthylph-thalamic acid | N/D | Solid phase extraction | - Calibration curve of all standards with ranges of 1.0-1000ng/mL, except for CDCA which was linear in the range 1.0-500ng/mL | N/D | - LOQ:   10ng/mL for CA, UDCA, and DCA.  2.5ng/mL for LCA  1.0ng/mL for TC and CDCA.   - LOD: N/D |
| [7] | Internal standard:  retinyl palmitate  Standards  Trans-retinol | Ethanol | Hexane | Standard curve | N/D | LOD: 0.316uM for retinol |
| [8]* | N/D | N/D | N/D | N/D | N/D | N/D |
| [9]* | Standards:  Kyn  Trp  ***Disclaimer:*** *No internal standards were used* | Perchloric acid | N/D | Calibration curve | N/D | LOD: 0.028 μmol/L for Kyn and 0.053 μmol/L for Trp |
| [10]* | Trp and Kyn standards were derivatized with to 30 μL of 100 mM sodium tetraborate and 50 μL of 2% benzoyl chloride-d5 (BzD5) to make an internal standard mixture labelled with BzD5-IS  Serum was spiked with sodium tetraborate and Bz. | N/D | N/D | Not well described | N/D | N/D |
| [11]* | Calibrations standards:   - L-Trp - D,L-Kyn - D,L-3-OH-Kyn   Internal standards for:   - L-Trp-2′,4′,5′,6′,7′-d5 - L-Kyn-3′,4′,5′,6′-d4 | N/D | Solid phase extraction | - Pooled quality controls spiked with calibration standards - Blank plasma samples obtained through dialysis of pooled plasma from HCs | N/D but calibrators were run with each batch. | - Calibration curve was done using serially diluted calibration curves. - LOD:30 nmol/L for Trp, 1 nmol/L for Kyn, and 5 nmol/L for 3-OH-Kyn - LOQ (at a signal-to-noise ratio of 10): 110 nmol/L for Trp, 50 nmol/L for Kyn, and 23 nmol/L nmol/L 3-OH-Kyn, with CVs of 13.4, 18.8, and 19.7%, respectively. |
| [12]* | Calibration standards:   - Trp - Kyn   Internal standards:   - Trp-d5 - Kyn-d4 | Trifluoroacetic acid | Charcoal strip | - Pooled quality controls spiked with standards. | N/D but calibrators were run | N/D |
| [13]* | Trp and Kyn standards were derivatized with to 30 μL of 100 mM sodium tetraborate and 50 μL of 2% benzoyl chloride-d5 (BzD5) to make an internal standard mixture labelled with BzD5-IS  Serum was spiked with sodium tetraborate and Bz. | N/D | N/D | Not well described | N/D | N/D |
| [14] | Internal standards:   - L-Phenylalanine-d8 - L-Tryptophan-d8 - L-Isoleucine-d10 - L-leucine-d10 - L-Methionine-d3 - L-Valine-d8 - L-Proline-d7 - L-Alanine-d7 - DL-Serine-d3 - DL-Glutamic acid-d5 - Glycine-d2 - L-Aspartic acid-d3 - L-Arginine-d7 - L-Glutamine-d5 - L-Lysine-d9 - L-Histidine-d5 - Taurine-13C2 - Betaine-d11 - Urea-(13C, 15N2) - L-lactate-d3 - Trimethylamine N-oxide-d9 - Choline-d13 - Malic acid-d3 - Citric acid-d4 - Succinic acid-d4 - Fumaric acid-d2 - Hypoxanthine-d3 - Xanthine-15N2 - Thymidine (13C10, 15N2) - Inosine-15N4, Uridine-d2 - Methylsuccinic acid-d6 - Benzoic acid-d5 - Creatine-d3 - Creatinine-d3 - Glutaric acid-d4 - Glycine-d2 - Kynurenic acid-d5 - L-Citrulline-d4 - L-Threonine-(13C4, 15N) - L-Tyrosine-d7 - P-cresol sulfate-d7 - Sarcosine-d3 - Trans-4-hydroxy-L-proline-d3 - Uric acid-(13C; 15N3)   ***Disclaimer:*** *For endogenous metabolites without labelled structural analogues, an automated algorithm selects the optimal internal standard for quantitation based on the rule of minimal coefficients of variations (CVs) after normalization.* | Methanol | N/D | - Quality controls were run every 10 injections | N/D | N/D |
| [15] | Biocrates kit with stable-isotope labelled internal standards | N/D | 300 µL of a 5 mM ammonium acetate in MeOH solution | - Pre-set quality control - Pre-set calibration curves | N/D | Was considered in analysis but is not well described. |

*****Method is published in other articles. Sitole, Tugizimana [3] cited [16]. Svensson Akusjärvi, Krishnan [4] cited [17] which cites another article doing untargeted metabolomics [18]. Chen, Xun [10] and Yang, Cai [13] cited [19]. Chen, Shao [9] cited [20]. [8] cited a review paper. Additionally, van der Ven, Blom [5] cites several articles [21-25] which was used for their methods.

Abbreviations: 3-OH-Kyn: 3-hydroxykynurenine; Bz: benzoyl chloride; BzD5: benzoyl chloride-d5; IS: internal standard; CA: cholic acid; CDCA: chenodeoxycholic acid; CV: coefficient of variation; DCA: deoxycholic acid; GC: gas chromatography; HC: healthy control; Kyn: kynurenine; LC: liquid chromatography; LCA: lithocholic acid; LOD: limit of detection; LOQ: limit of quantification; LPA: lysophosphatidic acid; MeOH: methanol; N/D: not described; QC: quality control; S/N: signal-to-noise; SPE: solid-phase extraction; TC: taurocholic acid; TFA: trifluoroacetic acid; Trp: tryptophan; UDCA: ursodeoxycholic acid; µM: micromolar; ng/mL: nanogram per millilitre; nmol/L: nanomoles per litre; kDa: kilodalton

**References**

1. Gebremicael G, Alemayehu M, Sileshi M, et al. The serum concentration of vitamin B(12) as a biomarker of therapeutic response in tuberculosis patients with and without human immunodeficiency virus (HIV) infection. *Int J Gen Med* 2019; 12: 353-361. DOI: 10.2147/ijgm.S218799

2. Kostadinova L, Shive CL, Judge C, et al. During Hepatitis C Virus (HCV) Infection and HCV-HIV Coinfection, an Elevated Plasma Level of Autotaxin Is Associated With Lysophosphatidic Acid and Markers of Immune Activation That Normalize During Interferon-Free HCV Therapy. *J Infect Dis* 2016; 214: 1438-1448. DOI: 10.1093/infdis/jiw372

3. Sitole LJ, Tugizimana F, Meyer D. Multi-platform metabonomics unravel amino acids as markers of HIV/combination antiretroviral therapy-induced oxidative stress. *J Pharm Biomed Anal* 2019; 176: 112796. DOI: 10.1016/j.jpba.2019.112796

4. Svensson Akusjärvi S, Krishnan S, Ambikan AT, et al. Role of myeloid cells in system-level immunometabolic dysregulation during prolonged successful HIV-1 treatment. *Aids* 2023; 37: 1023-1033. DOI: 10.1097/qad.0000000000003512

5. van der Ven AJ, Blom HJ, Peters W, et al. Glutathione homeostasis is disturbed in CD4-positive lymphocytes of HIV-seropositive individuals. *Eur J Clin Invest* 1998; 28: 187-193. DOI: 10.1046/j.1365-2362.1998.00267.x

6. McRae M, Rezk NL, Bridges AS, et al. Plasma bile acid concentrations in patients with human immunodeficiency virus infection receiving protease inhibitor therapy: possible implications for hepatotoxicity. *Pharmacotherapy* 2010; 30: 17-24. DOI: 10.1592/phco.30.1.17

7. Neves FF, Vannucchi H, Jordão AA, Jr., Figueiredo JF. Recommended dose for repair of serum vitamin A levels in patients with HIV infection/AIDS may be insufficient because of high urinary losses. *Nutrition* 2006; 22: 483-489. DOI: 10.1016/j.nut.2005.11.008

8. Baer SL, Colombo RE, Johnson MH, et al. Indoleamine 2,3 dioxygenase, age, and immune activation in people living with HIV. *J Investig Med* 2021; 69: 1238-1244. DOI: 10.1136/jim-2021-001794

9. Chen J, Shao J, Cai R, et al. Anti-retroviral therapy decreases but does not normalize indoleamine 2,3-dioxygenase activity in HIV-infected patients. *PLoS One* 2014; 9: e100446. DOI: 10.1371/journal.pone.0100446

10. Chen J, Xun J, Yang J, et al. Plasma Indoleamine 2,3-Dioxygenase Activity Is Associated With the Size of the Human Immunodeficiency Virus Reservoir in Patients Receiving Antiretroviral Therapy. *Clin Infect Dis* 2019; 68: 1274-1281. DOI: 10.1093/cid/ciy676

11. Jenabian MA, Patel M, Kema I, et al. Distinct tryptophan catabolism and Th17/Treg balance in HIV progressors and elite controllers. *PLoS One* 2013; 8: e78146. DOI: 10.1371/journal.pone.0078146

12. Somsouk M, Estes JD, Deleage C, et al. Gut epithelial barrier and systemic inflammation during chronic HIV infection. *Aids* 2015; 29: 43-51. DOI: 10.1097/qad.0000000000000511

13. Yang J, Cai R, Xun J, et al. Elevated indoleamine 2,3-dioxygenase activity is associated with endothelial dysfunction in people living with HIV and ROS production in human aortic endothelial cells in vitro. *Drug Discov Ther* 2023; 17: 312-319. DOI: 10.5582/ddt.2023.01069

14. Wan LY, Lam SM, Huang HH, et al. Multi-omics dissection of metabolic dysregulation associated with immune recovery in people living with HIV-1. *J Transl Med* 2025; 23: 143. DOI: 10.1186/s12967-025-06168-0

15. Cherenack EM, Larson ME, Murray K, et al. Stimulant Use, HIV, and Plasma Metabolites Among Men. *J Neuroimmune Pharmacol* 2025; 20: 68. DOI: 10.1007/s11481-025-10223-4

16. Mason S, Reinecke CJ, Solomons R. Cerebrospinal Fluid Amino Acid Profiling of Pediatric Cases with Tuberculous Meningitis. *Frontiers in Neuroscience* 2017; Volume 11 - 2017. DOI: 10.3389/fnins.2017.00534

17. Krishnan S, Nordqvist H, Ambikan AT, et al. Metabolic Perturbation Associated With COVID-19 Disease Severity and SARS-CoV-2 Replication. *Mol Cell Proteomics* 2021; 20: 100159. DOI: 10.1016/j.mcpro.2021.100159

18. Babu H, Sperk M, Ambikan AT, et al. Plasma Metabolic Signature and Abnormalities in HIV-Infected Individuals on Long-Term Successful Antiretroviral Therapy. *Metabolites* 2019; 9. DOI: 10.3390/metabo9100210

19. Cheng J, Jin H, Hou X, Lv J, Gao X, Zheng G. Disturbed tryptophan metabolism correlating to progression and metastasis of esophageal squamous cell carcinoma. *Biochem Biophys Res Commun* 2017; 486: 781-787. DOI: 10.1016/j.bbrc.2017.03.120

20. Zhang X, He Y, Ding M. Simultaneous determination of tryptophan and kynurenine in plasma samples of children patients with Kawasaki disease by high-performance liquid chromatography with programmed wavelength ultraviolet detection. *J Chromatogr B Analyt Technol Biomed Life Sci* 2009; 877: 1678-1682. DOI: 10.1016/j.jchromb.2009.04.013

21. Fiskerstrand T, Refsum H, Kvalheim G, Ueland PM. Homocysteine and other thiols in plasma and urine: automated determination and sample stability. *Clin Chem* 1993; 39: 263-271.

22. te Poele-Pothoff MT, van den Berg M, Franken DG, et al. Three different methods for the determination of total homocysteine in plasma. *Ann Clin Biochem* 1995; 32 ( Pt 2): 218-220. DOI: 10.1177/000456329503200218

23. Conti M, Morand PC, Levillain P, Lemonnier A. Improved fluorometric determination of malonaldehyde. *Clin Chem* 1991; 37: 1273-1275.

24. van der Ven AJ, Mier P, Peters WH, et al. Monochlorobimane does not selectively label glutathione in peripheral blood mononuclear cells. *Anal Biochem* 1994; 217: 41-47. DOI: 10.1006/abio.1994.1081

25. Fahey RC, Newton GL. Determination of low-molecular-weight thiols using monobromobimane fluorescent labeling and high-performance liquid chromatography. *Methods Enzymol* 1987; 143: 85-96. DOI: 10.1016/0076-6879(87)43016-4
