# Supplementary material for: Dysregulated Metabolism in People Living With HIV in the Modern ART‐Era: A Systematic Review of Targeted Metabolomics Studies
Source: Rev Med Virol. 2026 Jul 6;36(4):e70179. doi: 10.1002/rmv.70179 (PMC13335820; doi:10.1002/rmv.70179)
Supplement: Supplementary file 5 — Table S3: Stratification of the included studies according to sex. [file RMV-36-e70179-s010.docx]

**Supplementary Table 3:** Stratification of the included studies according to sex.

| Reference | Male | Mixed-Sex | Female | Metabolite Profile | |
| --- | --- | --- | --- | --- | --- |
|  |  |  |  | Dysregulated | No difference |
| [1]* |  | X |  | - Glutamine |  |
| [2] |  | X |  | - Glutamine - Kyn - Glycine - Ornithine |  |
| [3] | X |  |  | - Kyn/Trp |  |
| [4] | X |  |  |  | - Kyn - Kyn/Trp |
| [5] | X |  |  | - Kyn/Trp |  |
| [6] | X |  |  | - Kyn - Kyn/Trp |  |
| [7] | X |  |  | - Kyn/Trp |  |
| [8] | X |  |  |  | - Kyn - Kyn/Trp |
| [9] | X |  |  | - Glycine - Ornithine |  |

Effect sizes were reported without accompanying p-values in one some study [1].

*A subset of the cohort was used for targeted metabolomics but the sex distribution in the subset was not described though the total cohort consisted of mixed-sex.

Abbreviations: Kyn: kynurenine; Trp: tryptophan

1. Sitole LJ, Tugizimana F, Meyer D. Multi-platform metabonomics unravel amino acids as markers of HIV/combination antiretroviral therapy-induced oxidative stress. *J Pharm Biomed Anal* 2019; 176: 112796. DOI: 10.1016/j.jpba.2019.112796

2. Svensson Akusjärvi S, Krishnan S, Ambikan AT, et al. Role of myeloid cells in system-level immunometabolic dysregulation during prolonged successful HIV-1 treatment. *Aids* 2023; 37: 1023-1033. DOI: 10.1097/qad.0000000000003512

3. Baer SL, Colombo RE, Johnson MH, et al. Indoleamine 2,3 dioxygenase, age, and immune activation in people living with HIV. *J Investig Med* 2021; 69: 1238-1244. DOI: 10.1136/jim-2021-001794

4. Chen J, Shao J, Cai R, et al. Anti-retroviral therapy decreases but does not normalize indoleamine 2,3-dioxygenase activity in HIV-infected patients. *PLoS One* 2014; 9: e100446. DOI: 10.1371/journal.pone.0100446

5. Chen J, Xun J, Yang J, et al. Plasma Indoleamine 2,3-Dioxygenase Activity Is Associated With the Size of the Human Immunodeficiency Virus Reservoir in Patients Receiving Antiretroviral Therapy. *Clin Infect Dis* 2019; 68: 1274-1281. DOI: 10.1093/cid/ciy676

6. Jenabian MA, Patel M, Kema I, et al. Distinct tryptophan catabolism and Th17/Treg balance in HIV progressors and elite controllers. *PLoS One* 2013; 8: e78146. DOI: 10.1371/journal.pone.0078146

7. Somsouk M, Estes JD, Deleage C, et al. Gut epithelial barrier and systemic inflammation during chronic HIV infection. *Aids* 2015; 29: 43-51. DOI: 10.1097/qad.0000000000000511

8. Yang J, Cai R, Xun J, et al. Elevated indoleamine 2,3-dioxygenase activity is associated with endothelial dysfunction in people living with HIV and ROS production in human aortic endothelial cells in vitro. *Drug Discov Ther* 2023; 17: 312-319. DOI: 10.5582/ddt.2023.01069

9. Wan LY, Lam SM, Huang HH, et al. Multi-omics dissection of metabolic dysregulation associated with immune recovery in people living with HIV-1. *J Transl Med* 2025; 23: 143. DOI: 10.1186/s12967-025-06168-0

**References**
