# Supplementary material for: Dysregulated Metabolism in People Living With HIV in the Modern ART‐Era: A Systematic Review of Targeted Metabolomics Studies
Source: Rev Med Virol. 2026 Jul 6;36(4):e70179. doi: 10.1002/rmv.70179 (PMC13335820; doi:10.1002/rmv.70179)
Supplement: Supplementary file 7 — Table S5: ART information and HIV infection duration of the included studies. [file RMV-36-e70179-s006.docx]

**Supplementary Table 5:** ART information and HIV infection duration of the included studies.

| **Reference** | **ART Type** | **Regimen** | **Duration (months)** | **Duration of HIV-infection in months**  **Median (IQR)** |
| --- | --- | --- | --- | --- |
| [1] | HAART | N/D | Initiated at baseline – 6 months on HAART | N/D |
| [2] | ART | N/D | N/D | N/D |
| [3] | ART | N/D | N/D | N/D |
| [4] | ART | 3TC/DTG (NRTI/INSTI): 1.6%  ABC/3TC/DRV/ritonavir (NRTI/NRTI/PI/PI): 4.7%  ABC/3TC/DRV/Cob (NRTI/NRTI/PI/pharmokinetic booster): 1.6%  ABC/3TC/DTG (NRTI/NRTI/INSTI): 40.6%  ABC/3TC/EFV (NRTI/NRTI/NNRTI): 7.8%  ABC/3TC/NVP (NRTI/NRTI/NNRTI): 3.1%  ABC/3TC/RPV (NRTI/NRTI/NNRTI): 6.2%  CAB/RPV* (INSTI/NNRTI): 1.6%  DRV/COB/DTG (PI/PHARMOKINETIC BOOSTER/INSTI): 1.6%  RAL/3TC/EFV (INSTI/NRTI/NNRTI): 1.6%  TAF/FTC/DTG (NRTI/NRTI/INSTI): 9.3%  TAF/FTC/EFV: 1.6%  TDF/FTC/DTG (NRTI/NRTI/INSTI): 4.7%  TDF/FTC/EFV (NRTI/NRTI/NNRTI): 4.7%  TDF/FTC/EVG/Cob (NRTI/NRTI/INSTI/pharmokinetic booster): 1.6%  TDF/FTC/RPV (NRTI/NRTI/NNRTI): 7.8% | Duration of suppressive therapy: 7 (6-13) | 12 (9-20) years |
| [5] | ART: participant with <400 cells/mm^3^ | Regimen: not more than 2 reverse transcriptase inhibitors but the regimen was N/D | Duration: at least 3 months | N/D |
| [6] | HAART | Saquinavir (PI)  Ritonavir (PI)  Amprenavir-ritonavir (PI) | Initiated ART | N/D |
| [7] | 76% on HAART | N/D | N/D | N/D |
| [8] | ART | N/D | N/D | N/D |
| [9] | HAART | 2 NRTI+1 NNRTI: 96.1%  2 NRTI+1 PI: 3.9% | 1 year | N/D |
| [10] | ART | TDF + 3TC + EFV (NRTI/NRTI/NNRTI): 91.3%  ZDV + 3TC + EFV/NVP (NRTI/NRTI/NNRTI/NNRTI): 5.5%  TDF + 3TC + RAL (NRTI/NRTI/INSTI): 1.6%  TDF + 3TC + LPV/r (NRTI/NRTI/PI-booster): 1.6% | 15 (12-27) | N/D |
| [11] | ART | N/D | Duration: 8.1±4.7 [1-20] | 10.1±5.5 [2-24] |
| [12] | ART | N/D | INR: 11.3 (7.8-12) years  IR: 9.8 (4.4-10.7) years | N/D |
| [13] | ART | TDF+3TC+EFV (NRTI/NRTI/NNRTI): 84.2%  TDF+3TC+LPV/r (NRTI/NRTI/PI-booster): 5.3%  AZT+3TC+EFV (NRTI/NRTI/NNRTI): 7.9%  TDF+3TC+RAL (NRTI/NRTI/INSTI): 2.26% | 3 years | N/D |
| [14] | ART | Regimen for INRs:  NRTIs/NNRTIs: 100%  Regimen for IRs:  NRTIs/NNRTIs: 94%  NRTIs/PIs: 6% | INR: 45.6  (35.7–55.4) months  IR: 48.8  (23.8–118.3) months | N/D |
| [15] | ART | N/D | N/D | N/D |

Median (IQR); median ± SD; median (min, max); mean (SD); mean [range]; mean ± SD [range]

Abbreviations: N/D: Not described; 3TC: lamivudine; ABC: abacavir; ART: antiretroviral therapy; AZT; Azidothymidine (zidovudine); CAB: cabotegravir; COB: cobicistat; DRV: darunavir; DTG: dolutegravir; EFV: efavirenz; EVG: elvitegravir; FTC: emtricitabine; HAART: highly active antiretroviral therapy; HIV: human immunodeficiency virus; INSTI: integrase strand transfer inhibitor; INR: immunological non-responder; IQR: interquartile range; IR: immunological responder; LPV/r: lopinavir/ritonavir; NNRTI: non-nucleoside reverse transcriptase inhibitor; NRTI: nucleoside reverse transcriptase inhibitor; NVP: nevirapine; PI: protease inhibitor; RAL: raltegravir; RPV: rilpivirine; TAF: tenofovir alafenamide; TDF: tenofovir disoproxik fumarate; ZDV: zidovudine.

**References**

1. Gebremicael G, Alemayehu M, Sileshi M, et al. The serum concentration of vitamin B(12) as a biomarker of therapeutic response in tuberculosis patients with and without human immunodeficiency virus (HIV) infection. *Int J Gen Med* 2019; 12: 353-361. DOI: 10.2147/ijgm.S218799

2. Kostadinova L, Shive CL, Judge C, et al. During Hepatitis C Virus (HCV) Infection and HCV-HIV Coinfection, an Elevated Plasma Level of Autotaxin Is Associated With Lysophosphatidic Acid and Markers of Immune Activation That Normalize During Interferon-Free HCV Therapy. *J Infect Dis* 2016; 214: 1438-1448. DOI: 10.1093/infdis/jiw372

3. Sitole LJ, Tugizimana F, Meyer D. Multi-platform metabonomics unravel amino acids as markers of HIV/combination antiretroviral therapy-induced oxidative stress. *J Pharm Biomed Anal* 2019; 176: 112796. DOI: 10.1016/j.jpba.2019.112796

4. Svensson Akusjärvi S, Krishnan S, Ambikan AT, et al. Role of myeloid cells in system-level immunometabolic dysregulation during prolonged successful HIV-1 treatment. *Aids* 2023; 37: 1023-1033. DOI: 10.1097/qad.0000000000003512

5. van der Ven AJ, Blom HJ, Peters W, et al. Glutathione homeostasis is disturbed in CD4-positive lymphocytes of HIV-seropositive individuals. *Eur J Clin Invest* 1998; 28: 187-193. DOI: 10.1046/j.1365-2362.1998.00267.x

6. McRae M, Rezk NL, Bridges AS, et al. Plasma bile acid concentrations in patients with human immunodeficiency virus infection receiving protease inhibitor therapy: possible implications for hepatotoxicity. *Pharmacotherapy* 2010; 30: 17-24. DOI: 10.1592/phco.30.1.17

7. Neves FF, Vannucchi H, Jordão AA, Jr., Figueiredo JF. Recommended dose for repair of serum vitamin A levels in patients with HIV infection/AIDS may be insufficient because of high urinary losses. *Nutrition* 2006; 22: 483-489. DOI: 10.1016/j.nut.2005.11.008

8. Baer SL, Colombo RE, Johnson MH, et al. Indoleamine 2,3 dioxygenase, age, and immune activation in people living with HIV. *J Investig Med* 2021; 69: 1238-1244. DOI: 10.1136/jim-2021-001794

9. Chen J, Shao J, Cai R, et al. Anti-retroviral therapy decreases but does not normalize indoleamine 2,3-dioxygenase activity in HIV-infected patients. *PLoS One* 2014; 9: e100446. DOI: 10.1371/journal.pone.0100446

10. Chen J, Xun J, Yang J, et al. Plasma Indoleamine 2,3-Dioxygenase Activity Is Associated With the Size of the Human Immunodeficiency Virus Reservoir in Patients Receiving Antiretroviral Therapy. *Clin Infect Dis* 2019; 68: 1274-1281. DOI: 10.1093/cid/ciy676

11. Jenabian MA, Patel M, Kema I, et al. Distinct tryptophan catabolism and Th17/Treg balance in HIV progressors and elite controllers. *PLoS One* 2013; 8: e78146. DOI: 10.1371/journal.pone.0078146

12. Somsouk M, Estes JD, Deleage C, et al. Gut epithelial barrier and systemic inflammation during chronic HIV infection. *Aids* 2015; 29: 43-51. DOI: 10.1097/qad.0000000000000511

13. Yang J, Cai R, Xun J, et al. Elevated indoleamine 2,3-dioxygenase activity is associated with endothelial dysfunction in people living with HIV and ROS production in human aortic endothelial cells in vitro. *Drug Discov Ther* 2023; 17: 312-319. DOI: 10.5582/ddt.2023.01069

14. Wan LY, Lam SM, Huang HH, et al. Multi-omics dissection of metabolic dysregulation associated with immune recovery in people living with HIV-1. *J Transl Med* 2025; 23: 143. DOI: 10.1186/s12967-025-06168-0

15. Cherenack EM, Larson ME, Murray K, et al. Stimulant Use, HIV, and Plasma Metabolites Among Men. *J Neuroimmune Pharmacol* 2025; 20: 68. DOI: 10.1007/s11481-025-10223-4
